# Cross-species metabolomic analysis identifies uridine as a potent regeneration promoting factor

**DOI:** 10.1038/s41421-021-00361-3

**Published:** 2022-02-01

**Authors:** Zunpeng Liu, Wei Li, Lingling Geng, Liang Sun, Qiaoran Wang, Yang Yu, Pengze Yan, Chuqian Liang, Jie Ren, Moshi Song, Qian Zhao, Jinghui Lei, Yusheng Cai, Jiaming Li, Kaowen Yan, Zeming Wu, Qun Chu, Jingyi Li, Si Wang, Chunyi Li, Jing-Dong J. Han, Reyna Hernandez-Benitez, Ng Shyh-Chang, Juan Carlos Izpisua Belmonte, Weiqi Zhang, Jing Qu, Guang-Hui Liu

**Affiliations:** 1 State Key Laboratory of Stem Cell and Reproductive Biology, Institute of Zoology, Chinese Academy of Sciences, Beijing, China; 2 Institute for Stem cell and Regeneration, CAS, Beijing, China; 3 University of Chinese Academy of Sciences, Beijing, China; 4 Advanced Innovation Center for Human Brain Protection, National Clinical Research Center for Geriatric Disorders, Xuanwu Hospital Capital Medical University, Beijing, China; 5 Aging Translational Medicine Center, Xuanwu Hospital, Capital Medical University, Beijing, China; 6 The Key Laboratory of Geriatrics, Beijing Institute of Geriatrics, Institute of Geriatric Medicine, Chinese Academy of Medical Sciences, Beijing Hospital/National Center of Gerontology of National Health Commission, Beijing, China; 7 The NHC Key Laboratory of Drug Addiction Medicine, Kunming Medical University, Kunming Yunnan, China; 8 CAS Key Laboratory of Genomic and Precision Medicine, Beijing Institute of Genomics, Chinese Academy of Sciences, Beijing, China; 9 China National Center for Bioinformation, Beijing, China; 10 Beijing Key Laboratory of Reproductive Endocrinology and Assisted Reproductive Technology and Key Laboratory of Assisted Reproduction, Department of Obstetrics and Gynecology, Ministry of Education, Center for Reproductive Medicine, Peking University Third Hospital, Beijing, China; 11 State Key Laboratory of Membrane Biology, Institute of Zoology, Chinese Academy of Sciences, Beijing, China; 12 Beijing Institute for Stem Cell and Regenerative Medicine, Beijing, China; 13 Institute of Antler Science and Product Technology, Changchun Sci-Tech University, Changchun Jilin, China; 14 Peking-Tsinghua Center for Life Sciences, Academy for Advanced Interdisciplinary Studies, Center for Quantitative Biology (CQB), Peking University, Beijing, China; 15 Gene Expression Laboratory, Salk Institute for Biological Studies, La Jolla, CA USA

**Keywords:** Ageing, Stem cells

## Abstract

Regenerative capacity declines throughout evolution and with age. In this study, we asked whether metabolic programs underlying regenerative capability might be conserved across species, and if so, whether such metabolic drivers might be harnessed to promote tissue repair. To this end, we conducted metabolomic analyses in two vertebrate organ regeneration models: the axolotl limb blastema and antler stem cells. To further reveal why young individuals have higher regenerative capacity than the elderly, we also constructed metabolic profiles for primate juvenile and aged tissues, as well as young and aged human stem cells. In joint analyses, we uncovered that active pyrimidine metabolism and fatty acid metabolism correlated with higher regenerative capacity. Furthermore, we identified a set of regeneration-related metabolite effectors conserved across species. One such metabolite is uridine, a pyrimidine nucleoside, which can rejuvenate aged human stem cells and promote regeneration of various tissues in vivo. These observations will open new avenues for metabolic intervention in tissue repair and regeneration.

## Introduction

Regeneration is the process of rejuvenating or replacing damaged, diseased, or aged tissues^1^. From lower animals to humans, every species is endowed with a certain degree of regeneration. For example, axolotl, the Mexican salamander, or the “walking fish”, is evolutionarily primitive vertebrate known to possess a higher regenerative capacity than mammals^2–5^. Another example is the deer antler, which is the only organ capable of complete regeneration in mammals^6–8^. In most mammals, the limited anatomical and functional recovery capabilities reside in young tissue and decline with age^2,9–11^, leading to compromised tissue repair after injury.

Across species, stem cells usually take center stage in tissue repair and regeneration^1,12^. The axolotl can regenerate their limbs through the formation of blastema tissue, a mass of dedifferentiated stem cells^3,5,13^. Similarly, during annual regeneration, deer antler produces a whole organ containing blood vessels, cartilage, bone, dermis, and nerves from deer antler stem cells (dASCs) that express classic mesenchymal stem cell (MSC) markers and reside in the mid-beam antler periosteum^7,14–16^. Compared to stem cells from regenerative tissues of the axolotl limb and the deer antler, human stem cells, such as human mesenchymal stem cells (hMSCs), possess a relatively limited capacity for regenerative repair of damages to vital tissues and organs^2,12^, but gradually lose such capacity with age. Whether molecular characteristics between these naturally occurring regeneration processes are evolutionarily conserved across species is unknown.

Using comparative methods to describe the similarities and differences between species is a powerful strategy to discover the regulatory mechanisms that underline vital life events, such as regeneration. The effectiveness of this method depends on whether there are comparable and sufficient overlapping factors across different samples. Unlike proteins that are biomacromolecules, the structure of metabolites is relatively similar between species, making metabolism an ideal research area for investigating evolutionarily conserved biology^17^. Yet, a systematic and high-resolution metabolomic characterization across paradigms with high regenerative capacity in different species and different tissues has not yet been attempted. The rapid development of untargeted metabolomic profiling could serve this purpose^18^.

Here, we sought to understand how metabolic regulation intersects with inherent regenerative capacity using comparative approaches. Samples for this study included i) species that are more primitive on the evolutionary scale but can renew entire organs, and ii) higher species in evolution that have lost full organ regenerative capacity but retain a limited capacity for tissue repair. We systematically depicted metabolic profiles in various regeneration-related contexts, and we discovered that high pyrimidine and fatty acid metabolism was shared across species, tissues, and cells with high regenerative capacity. We identified uridine as a pro-regenerative metabolite that promoted human stem cell activity and enhanced regeneration in multiple tissues in mammals.

## Results

### Transcriptomic analysis revealing convergent metabolic pathways in models with enhanced regenerative potentials

**Fig. 1:**
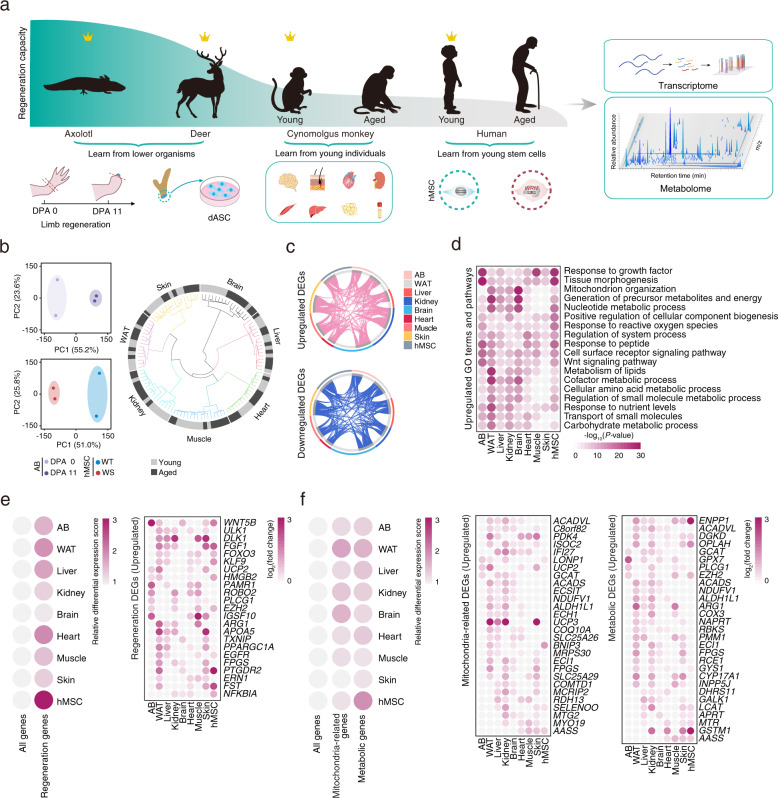
Cross-species transcriptomic features associated with differential regenerative capacities. **a** Flowchart of experimental design for obtaining transcriptome and metabolome data from samples with differential regenerative capacities: axolotl blastema (AB) at DPA 0 and DPA 11, deer antler stem cells (dASCs), tissues from young and aged non-human primates (NHPs), and young and aged hMSCs. **b** Left, PCA analysis of the transcriptome data of AB at DPA 0 and DPA 11 (top) and WT and WS hMSCs (bottom). Right, tree plot showing the Euclidean distance for transcriptome data of young and aged NHP tissues. **c** Circos plots showing the overlap of upregulated (top) and downregulated (bottom) DEGs in AB at DPA 11, young tissues, and young hMSCs. **d** Bubble plot showing the enriched GO terms and pathways for upregulated DEGs in AB at DPA 11, young tissues, and young hMSCs. The color key from white to amaranth indicates low to high –log_10_(*P*-value). **e** Left, bubble plot showing the relative differential expression (DE) score for all genes and regeneration genes in AB at DPA 11, young tissues, and young hMSCs. The color key from white to amaranth indicates the relative DE scores from low to high. Right, bubble plot showing the convergently upregulated regeneration DEGs in AB at DPA 11, young tissues, and young hMSCs. The color key from white to amaranth indicates log_2_(fold change) values of DEGs from low to high. Genes convergently upregulated in at least four tissues/cells with high regenerative capacity were shown. **f** Left, bubble plot showing the relative DE score for all genes, mitochondria-related genes or metabolic genes in AB at DPA 11, young tissues, and young hMSCs. Middle, bubble plot showing the convergently upregulated mitochondria-related DEGs in AB at DPA 11, young tissues, and young hMSCs. Right, bubble plot showing the convergently upregulated metabolic DEGs in AB at DPA 11, young tissues, and young hMSCs. The color key from white to amaranth indicates DE scores (left) or log_2_(fold change) values of DEGs (middle and right) from low to high. Genes convergently upregulated in at least four tissues/cells with higher regenerative capacity were shown.

We hypothesized that comparative studies of highly divergent regenerative models might reveal evolutionarily conserved programs driving tissue regeneration^2,19^. To test this hypothesis, we decided to profile the transcriptomes and metabolomes of samples from naturally occurring regeneration processes in vertebrate organ regeneration, and young and old primate tissues and human stem cell models with differential regenerative capacities^20^ (Fig. 1a). To represent whole organ regeneration, we chose the axolotl limb and deer antler. We obtained axolotl blastema (AB) at the amputation surface at day 11 post amputation (DPA 11), the timepoint when axolotl blastema stem cells (aBSCs) peak^21^ (Fig. 1a). Similarly, we isolated dASCs from the amputation surface. dASCs have a higher self-renewal ability than human primary mesenchymal stem cells (hPMSCs)^6^^,7,16^ (Fig. 1a and Supplementary Fig. S1a–c). In human stem cell model, young wild-type hMSCs manifested higher self-renewal and regenerative abilities relative to prematurely aged control (WRN-depleted hMSCs, mimicking Werner syndrome (WS), a human progeroid syndrome)^22–27^ (Fig. 1a and Supplementary Fig. S1d–f). To represent young tissues, we obtained eight tissues/organs from young and old non-human primates (NHPs) for comparison. The tissues (i.e., liver, skeletal muscle, skin, kidney, brain, heart, white adipose tissue (WAT), as well as blood plasma) selected for further analysis also hold varied regenerative abilities (Fig. 1a).

Genome-wide RNA sequencing analysis revealed that differentially expressed genes (DEGs) overlapped extensively across axolotl and NHP tissues, and genes involved in regeneration-related Gene Ontology (GO) terms or pathways, including “response to growth factor” and “tissue morphogenesis” were upregulated in axolotl blastema at DPA 11 and young NHP tissues (Fig. 1b–d and Supplementary Fig. S1g–h). Interestingly, the upregulated DEGs were convergently enriched in metabolism-related terms, including “mitochondrial organization”, “generation of precursor metabolites and energy” and “nucleotide metabolic process” (Fig. 1d and Supplementary Fig. S1h).

As expected, convergently upregulated DEGs, included known regeneration-related genes, such as growth factors or transcriptional factors required for organismal development (Fig. 1e). Of note, *PPARGC1A*, the master gene of mitochondrial biogenesis and fatty acid metabolism, was upregulated in most of the young tissues (Fig. 1e). Concomitantly, we also observed convergent upregulation of mitochondrial and metabolic pathways in tissues/cells with higher regenerative capacity (Fig. 1f). Among them, *ACADVL*, *ACADS*, *ECH1*, and *ECI1* are all enzymes that catalyze fatty acid oxidation (FAO) in mitochondria (Fig. 1f). For example, *ACADVL*, the dehydrogenase that catalyzes the first step of mitochondrial FAO^28,29^, was upregulated in most of the young tissues (Fig. 1f). These results imply that metabolic regulation is an important feature associated with regenerative potential, and suggest that our framework approach may unveil metabolic commonalities across species and tissues.

### Metabolomic analysis unveiling conservation and difference in metabolic characteristics between species

**Fig. 2:**
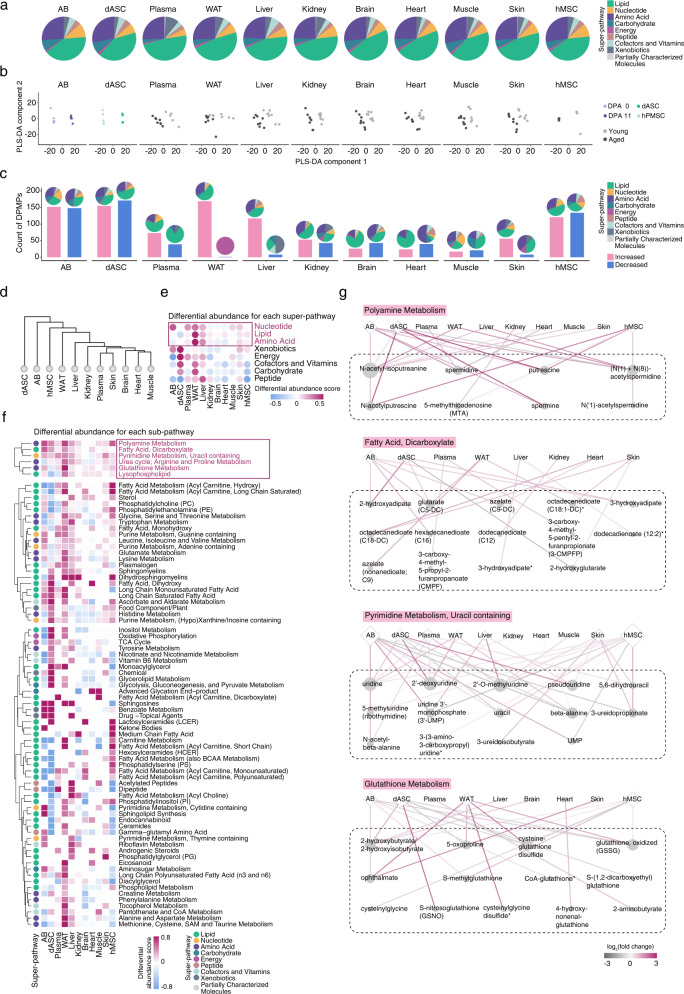
Cross-species metabolomic features underlying differential regenerative capacities. **a** Pie plot showing the percentage of super-pathway for identified metabolites in AB, dASCs, NHP tissues and hMSCs. **b** PLS-DA analysis of metabolomic data generated in AB, dASCs, NHP tissues and hMSCs. **c** Bar plot showing the count of DPMPs identified in AB at DPA 11, dASCs, young NHP tissues, and young hMSCs. The percentages of DPMPs in each super-pathway were shown on the top of each bar graph. **d** The hierarchical clustering dendrogram showing the similarity of the metabolic profile changes in AB at DPA 11, dASCs, young NHP tissues, and young hMSCs. **e** Heatmap showing the differential abundance (DA) for identified metabolites in each super-pathway in AB at DPA 11, dASCs, young NHP tissues, and young hMSCs. The color key from blue to amaranth indicates DA score from low to high for each super-pathway. **f** Heatmap showing the differential abundance for identified metabolites in each sub-pathway in AB at DPA 11, dASCs, young NHP tissues, and young hMSCs. Sub-pathways increased in at least seven tissues/cells with higher regenerative capacity were highlighted. The color key from blue to amaranth indicates DA score from low to high for each sub-pathway. **g** Network diagram showing the representative sub-pathways and relative abundance for DPMPs in each sub-pathway. The color of the edge from grey to amaranth indicates log_2_(fold change) from low to high. The node sizes are positively correlated to the edge counts for each node.

Metabolic processes are fundamental for organismal growth and development, and are required for the coordinated regulation of regeneration^30,31^. Indeed, we found that the convergent changes of metabolism-related genes across species were more prominent than that of global gene expression between samples with differential regeneration capacities (Fig. 1f). Next, we employed ultrahigh performance liquid chromatography-tandem mass spectroscopy (UPLC-MS/MS)-based metabolomics to profile metabolites across our models with differential regenerative abilities (Fig. 1a). After stringent quality control and normalization, we identified a range of 400–759 metabolites of various classes (including lipids, nucleotides, amino acids, carbohydrates, energy, peptides, cofactors, and vitamins) in each of the phylogenetically distant models (Fig. 2a and Supplementary Fig. S2a). However, we found that the metabolite distributions segregating with higher regenerative capacity were overall comparable across the axolotl blastema, dASCs, eight tissues of NHPs, and human stem cells (Fig. 2a), indicative of a considerable level of evolutionary conservation.

To characterize metabolic features in samples with higher regenerative potential, we performed partial least squares discriminant analysis (PLS-DA) (Fig. 2b). Overall, our analysis showed a clear separation of metabolomes between samples with higher regenerative abilities and their control counterparts (Fig. 2b and Supplementary Fig. S2b), indicating a clear correlation between metabolic features and regenerative capacities. Next, we assessed the changes of metabolite abundance underlying differential regenerative potentials and identified differentially present metabolic products (DPMPs) (Fig. 2c). Globally, metabolic profiles were most influenced by species diversity, followed by tissue specificity (Fig. 2c, d). In addition, WAT had the most distinct metabolic changes out of all the tested tissues between old and young individuals, highlighting the uniqueness of WAT metabolism (Fig. 2c, d and Supplementary Fig. S2b). In all, these analyses demonstrate that metabolomic profiles of samples with higher regenerative potentials are clearly different from their less regenerative counterparts.

### Identification of key metabolites associated with higher regenerative potential

Next, we assigned DPMPs to super-pathways and sub-pathways according to metabolite annotations (Fig. 2e, f). At the super-pathway level, lipids, amino acids, and nucleotides account for ~60% of metabolic changes in all models (Fig. 2c, e). In general, nucleotide metabolism appeared more prominent in the blastema and young NHP tissues, while lipid metabolism was highly abundant in almost all young NHP tissues and young hMSCs (Fig. 2e).

Within the super-pathways, we were intrigued to see a metabolite subset uniformly more abundant in almost all samples with higher regenerative potential than their regeneration-refractory counterparts (Fig. 2f). For example, uracil-containing pyrimidine metabolites in the nucleotide super-pathway were enriched in the blastema, dASCs, young WT-hMSCs, and almost all young NHP samples (Fig. 2f, g). Guanine-containing purine metabolites were abundant in the blastema, young WT-hMSCs, young NHP plasma, WAT, and liver (Fig. 2f). Pyrimidine and purine metabolism offer structural blocks for DNA and RNA synthesis, critical to vital biological processes, including development^32^. Metabolites in lipid metabolism sub-pathways, such as fatty acid (dicarboxylate) and lysophospholipid, were predominantly enriched in almost all samples (Fig. 2f, g, and Supplementary Fig. S2c), reflecting the metabolic potential of lipolysis in models with higher regenerative capacity. In addition, long-chain mono-unsaturated/saturated fatty acids, phosphatidylcholine (PC), and phosphatidylethanolamine (PE) were also substantially enriched in most of the young tissues or stem cells (Fig. 2f).

Four sub-pathways related to amino acid metabolism, namely, polyamine metabolism, urea cycle/arginine and glutathione metabolism, glycine, serine and threonine metabolism, were also enriched (Fig. 2f, g). Among these, glutathione metabolism plays an important role in defensing against oxidant^33,34^, and was recently reported to be activated in liver regeneration^35^. In addition, polyamine metabolites (e.g., spermine and spermidine) are well-known pro-regenerative metabolites^36–38^. Taken together, these data imply a metabolic preference underlying high regenerative ability.

### Transcriptional regulation of metabolic pathways related to regenerative capacity

To dissect the identified regulatory pathways, we leveraged the genome-wide transcriptomic analysis with a focus on metabolic genes and conducted an integrated pathway-level analysis of transcriptomic and metabolomic data with the MetaboAnalyst web tool^39^ (Supplementary Fig. S3a). This analysis revealed that within each super-pathway, specific sub-pathways were activated in almost all models, i.e., “pyrimidine metabolism” in the nucleotide super-pathway, “glycerophospholipid metabolism”, “glycerol metabolism” and “fatty acid degradation” in the lipid super-pathway, and “glutathione metabolism” in the amino acid super-pathway (Supplementary Fig. S3a). In pyrimidine metabolism, both DPMPs (uridine and 2′-deoxyuridine) and differentially expressed metabolic genes (DEMGs) (i.e., *UCK2*, *UCKL1*, and *RRM1*) were highly represented in samples with higher regenerative potentials (Supplementary Fig. S3b).

Transcriptional regulatory network analysis of DEMGs further underscored that the regulons underlying metabolic regulation overlap across models (Supplementary Fig. S3c, d). Notably, almost all PPAR-RXR complex members were identified as core transcription factors, namely *PPARA*, *PPARD*, *PPARG*, and their coactivator *PPARGC1A*, as well as the retinoid X receptors (*RXRA* and *RXRG*) (Supplementary Fig. S3c, d). As one of the most prominent regulatory systems for maintaining lipid and glucose homeostasis, the PPAR-RXR complex regulates a suite of genes involved in lipid and nucleotide metabolic processes^40–42^. Consistently, we found that the genes involved in the PPAR signaling pathway were activated, including those involved in lipid transport (*APOA1*, *APOA2*, *APOC3*, and *APOA5*) and fatty acid transport (*ACSL4* and *SLC27A1*) (Supplementary Fig. S3e). Additionally, the molecular program for FAO was also more active in samples with higher regenerative potentials, including mitochondrial genes essential for the uptake of long-chain fatty acids (*CPT1* and *CPT2*) (Supplementary Fig. S3e). Overall, the combined metabolomic and transcriptomic datasets revealed a strong co-occurrence between lipid and pyrimidine metabolism and identified the PPAR-RXR complex as a potential hub to be involved in the regulation of regeneration.

### Screening of natural metabolites reinforcing hMSC activity

**Fig. 3:**
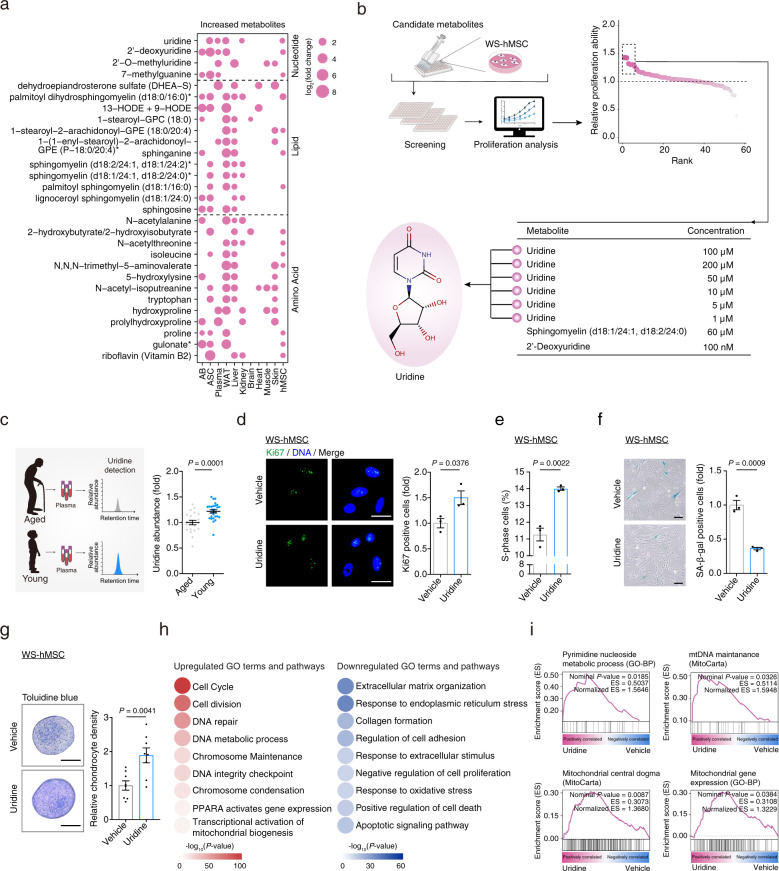
Uridine treatment promotes hMSC activity. **a** Bubble plot showing the increased metabolites in at least four tissues/cells with higher regenerative capacity. The bubble sizes are positively correlated to the log_2_(fold change) values. **b** Top-left, schematic illustration of the screening strategy for candidate metabolites reinforcing self-renewal activity of WS hMSCs. Top-right, scatter plot showing the relative cell proliferative abilities upon treatment with candidate metabolites at indicated concentrations in WS hMSCs. Bottom, top-ranked metabolites at indicated concentrations for cell proliferation are shown in the table. **c** Schematic representation (left) and quantitative data (right) of the detection of uridine concentration in the plasma of young (19–25 years old, *n* = 28) and aged (75–92 years old, *n* = 21) individuals. **d** Immunostaining of Ki67 in vehicle- and uridine (200 μM)-treated WS hMSCs (passage 5, P5) at P2 post treatment. Data are presented as the mean ± SEM (two-tailed unpaired Student’s *t* test). *n* = 3 biological replicates. Scale bars, 25 μm. **e** Cell cycle analysis of vehicle- and uridine (200 μM)-treated WS hMSCs (P5) at P2 post treatment. Data are presented as the mean ± SEM (two-tailed unpaired Student’s *t* test). *n* = 3 biological replicates. **f** SA-β-gal staining of vehicle- and uridine (200 μM)-treated WS hMSCs (P5) at P2 post treatment. Scale bars, 100 μm. Data are presented as the mean ± SEM (two-tailed unpaired Student’s *t* test). *n* = 3 biological replicates. **g** Toluidine blue staining analysis to evaluate the chondrogenesis of vehicle- and uridine (200 μM)-treated WS hMSCs (P5) at P2 post treatment. Data are presented as the mean ± SEM (two-tailed unpaired Student’s *t* test). *n* = 8 biological replicates. Scale bars, 100 μm. **h** Heatmap diagrams showing enriched GO terms and pathways for upregulated genes (left) and downregulated genes (right) in uridine (200 μM)-treated WS hMSCs (P5) at P2 post treatment as compared to vehicle-treated counterparts. The color keys from white to red or blue indicate the enrichment levels [–log_10_(*P*-value)] from low to high. **i** Gene set enrichment analysis (GSEA) showing representative GO terms and pathways in uridine (200 μM)-treated WS hMSCs (P5) at P2 post treatment as compared to vehicle-treated counterparts.

We next asked if it was possible to identify a metabolite that could boost the activity of human stem cells. To answer this question, we identified 29 candidate metabolites as upregulated DPMPs in at least four tissues or cells with higher regenerative ability from the nucleotide, amino acid, and lipid super-pathways (Fig. 3a and Supplementary Table S1). Among them, palmitoyl dihydrosphingomyelin (d18:0/16:0), lignoceroyl sphingomyelin (d18:1/24:0), sphingomyelin (d18:1/24:1, d18:2/24:0), and sphingosine in lipid super-pathway and uridine, 2′-deoxyuridine, 2′-O-methylguanine, and 7-methylguanine in nucleotide super-pathway were enriched in at least 4 tissues or cells with a higher regenerative ability (Fig. 3a).

From this set of candidates, we screened commercially available metabolites (Supplementary Table S2) for their effects in promoting self-renewal of aged hMSCs (Fig. 3b), which is based on the known fact that regenerative ability in hMSCs is correlated with their self-renewal activity^22^^,43^^,44^. In line with the activated lipid metabolism in young hMSCs, sphingomyelin (d18:1/24:1, d18:2/24:0) supplementation stimulated hMSC self-renewal (Fig. 3a, b). More importantly, uridine, which we found to be more abundant in the plasma from young individuals than that from old individuals (Fig. 3c), was identified as a metabolite segregating with higher activity in WS hMSCs (Fig. 3b).

When we supplemented the culture medium with uridine, we found that uridine treatment was sufficient to reprogram the prematurely and physiologically aged stem cell models (WS/HGPS (Hutchinson-Gilford progeria syndrome)-hMSCs and hPMSCs) into a younger state with a higher regenerative ability (Fig. 3d–f and Supplementary Fig. S4a–f). Specifically, uridine-treated hMSCs achieved a much higher proliferation rate and an enhanced capacity to form cartilage and gained increased genome and epigenome stability (Fig. 3d–g and Supplementary Fig. S4a–f). In accordance, genome-wide RNA-seq analysis showed that upregulated genes were mainly associated with “cell cycle” and “DNA integrity checkpoint” GO terms or pathways (Fig. 3h). Consistent with a previous study reporting that uridine addition rescues pyrimidine biosynthesis deficiency^45^, we found that the “pyrimidine nucleoside metabolic process” was elevated by uridine supplementation (Fig. 3i). Uridine treatment also appears to have a beneficial role in mitochondrial activity, as we found augmented gene expression associated with “mitochondrial central dogma”, “mtDNA maintenance”, and “mitochondrial gene expression” in uridine-treated hMSCs (Fig. 3i). Taken together, these results showed that uridine supplementation drives broad transcriptional changes associated with improved hMSC activity.

### Uridine treatment enhances regeneration and repair in various types of tissues

**Fig. 4:**
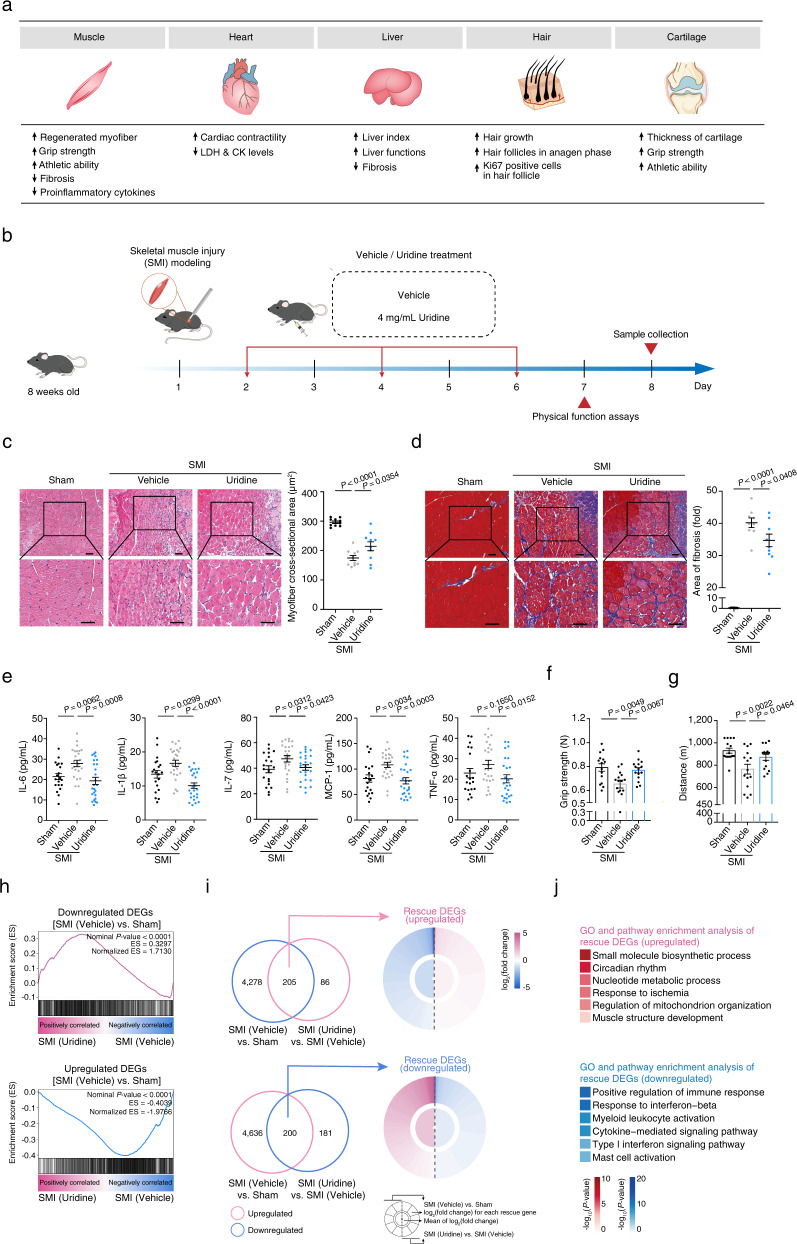
Uridine enhances muscle regeneration in vivo. **a** Schematic diagram showing the summarized phenotypes in tissue regeneration or repair models. **b** Schematic diagram for the time course of the mouse muscle cryoinjury and vehicle or uridine treatment. **c** Left, haematoxylin and eosin (H&E) staining of the skeletal muscle derived from sham mice (*n* = 10 mice) and mice treated with vehicle (*n* = 10 mice) or uridine (*n* = 10 mice) post cryoinjury. Right, quantitative data of mean myofiber cross-sectional area (CSA) in the skeletal muscle derived from sham mice and mice treated with vehicle or uridine post cryoinjury. Data are presented as the means ± SEM (two-tailed unpaired Student’s *t-*test). Scale bars, 100 μm. **d** Left, Masson staining of the quadriceps femoris derived from sham mice (*n* = 9 mice) and injured mice treated with vehicle (*n* = 9 mice) or uridine (*n* = 9 mice). Right, quantitative data of the fibrotic area. Data are presented as the means ± SEM (two-tailed unpaired Student’s *t-*test). Scale bars, 100 μm. **e** ELISA detecting the secretion of proinflammatory factors in the serum of sham mice (*n* = 20 mice) and mice treated with vehicle (*n* = 25 mice) or uridine (*n* = 25 mice) post cryoinjury. Data are presented as means ± SEM (two-tailed unpaired Student’s *t-*test). **f** Grip strength evaluation of the hind limbs of sham mice (*n* = 15 mice) and mice treated with vehicle (*n* = 15 mice) or uridine (*n* = 15 mice) at day 7 post cryoinjury. Data are presented as means ± SEM (two-tailed unpaired Student’s *t* test). **g** Treadmill distance of sham mice (*n* = 15 mice) and mice treated with vehicle (*n* = 15 mice) or uridine (*n* = 15 mice) at day 7 post cryoinjury. Data are presented as means ± SEM (two-tailed unpaired Student’s *t-*test). **h** Gene set enrichment analysis showing relative expression levels for downregulated DEGs upon cryoinjury (top) and upregulated DEGs upon cryoinjury (bottom) in the muscle tissues derived from vehicle- or uridine-treated mice. **i** Top, Venn diagram showing the downregulated genes upon cryoinjury and upregulated genes upon uridine treatment as compared to vehicle treatment. The overlapped genes were defined as “rescue DEGs (upregulated)” (left). Ring-heatmap plot showing the relative expression levels of “rescue DEGs (upregulated)” in mouse muscle regeneration model (right). Bottom, Venn diagram showing the upregulated genes upon cryoinjury and downregulated genes upon uridine treatment as compared to vehicle treatment. The overlapped genes were defined as “rescue DEGs (downregulated)” (left). Ring-heatmap plot showing the relative expression levels of “rescue DEGs (downregulated)” in mouse muscle regeneration model (right). The color key from blue to amaranth indicates log_2_(fold change) values from low to high. **j** Top, GO term and pathway enrichment analysis of “rescue DEGs (upregulated)” in mouse muscle regeneration model. Bottom, GO term and pathway enrichment analysis of “rescue DEGs (downregulated)” in mouse muscle regeneration model. The color keys from white to red or blue indicate –log_10_(*P*-value) from low to high.

**Fig. 5:**
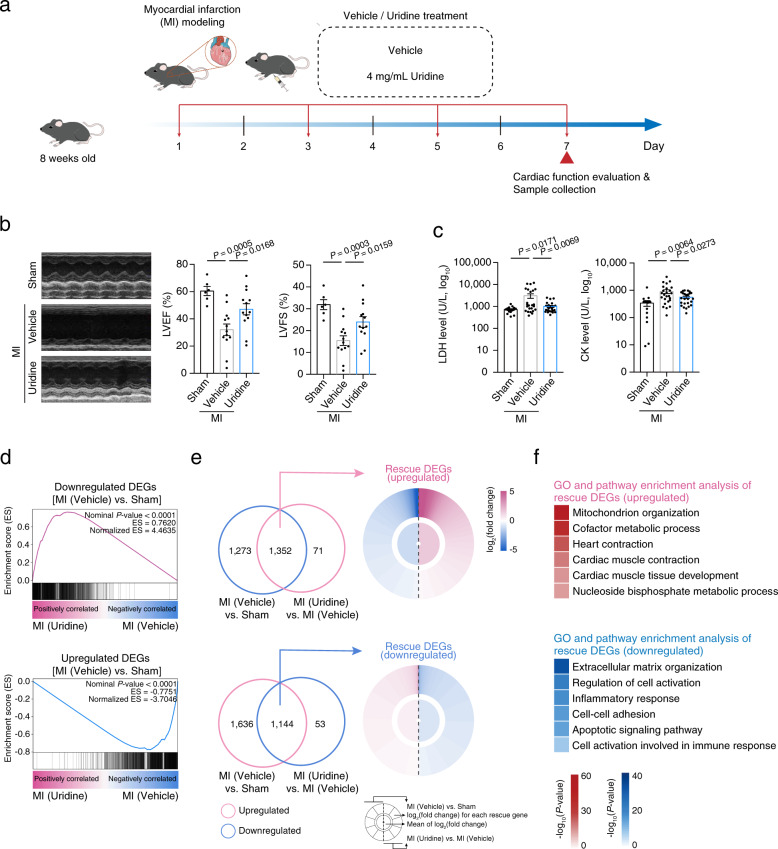
Uridine promotes cardiac repair after myocardial infarction. **a** Schematic diagram for the time course of the mouse myocardial infraction (MI) modeling and vehicle or uridine treatment. Uridine or vehicle treatment were performed every other day, as indicated by the red arrows. **b** Left, representative echocardiographic images of sham mice (*n* = 6 mice) and mice treated with vehicle (*n* = 13 mice) or uridine (*n* = 13 mice) at day 7 after MI modeling and intramyocardial injection of vehicle or uridine. Right, quantitative data of left ventricular ejection fraction (LVEF) and left ventricular fractional shortening (LVFS). Data are presented as the means ± SEM (two-tailed unpaired Student’s *t-*test). **c** Quantitative analysis of LDH and CK level of sham mice (*n* = 15 mice) and mice treated with vehicle (*n* = 25 mice) or uridine (*n* = 25 mice) on the next day after MI modeling and intramyocardial injection of vehicle or uridine. Data are presented as the means ± SEM (two-tailed unpaired Student’s *t-*test). **d** Gene set enrichment analysis showing relative expression levels for downregulated DEGs upon myocardial infarction (top) and upregulated DEGs upon myocardial infarction (bottom) in the heart tissues from vehicle- or uridine-treated mice. **e** Top, Venn diagram showing the downregulated genes upon myocardial infarction and upregulated genes upon uridine treatment as compared to vehicle treatment. The overlapped genes were defined as “rescue DEGs (upregulated)” (left). Ring-heatmap plot showing the relative expression levels of “rescue DEGs (upregulated)” in mouse myocardial infarction model (right). Bottom, Venn diagram showing the upregulated genes upon myocardial infarction and downregulated genes upon uridine treatment as compared to vehicle treatment. The overlapped genes were defined as “rescue DEGs (downregulated)” (left). Ring-heatmap plot showing the relative expression levels of “rescue DEGs (downregulated)” in mouse myocardial infarction model (right). The color key from blue to amaranth indicates log_2_(fold change) values from low to high. **f** Top, GO term and pathway enrichment analysis of “rescue DEGs (upregulated)” in mouse myocardial infarction model. Bottom, GO term and pathway enrichment analysis of “rescue DEGs (downregulated)” in mouse myocardial infarction model. The color keys from white to red or blue indicate –log_10_(*P*-value) from low to high.

The extent of tissue repair after injury is limited by organismal intrinsic regenerative capacity^46^. Next, we asked whether uridine supplementation could promote regeneration or tissue repair in multiple tissues, including skeletal muscle, heart, liver, skin, and articular cartilage (Fig. 4a). Relative to vehicle-treated mice, we observed that uridine treatment promoted tissue repair in both muscular and cardiac injury models (Figs. 4b–j, 5a–f, and Supplementary Fig. S5a–d). For instance, uridine treatment facilitated muscle tissue regeneration, reduced fibrotic or erosion area, decreased proinflammatory cytokine levels, and endowed treated mice with higher grip strength and longer running distance (Fig. 4b–g). We next performed genome-wide RNA-seq analysis in injured muscles with or without uridine treatment (Fig. 4h–j). In line with the decreased levels of proinflammatory cytokines in mouse serum in the uridine-treated groups (Fig. 4e), bulk RNA sequencing showed uridine supplementation antagonized the expression of a panel of the inflammatory genes, the expression of which was elevated in injured muscles (Fig. 4j). In comparison, pathways related to muscle structure development, as well as metabolic pathways, especially in “small-molecule biosynthetic process” and “nucleotide metabolic process” were upregulated in uridine-treated mice (Fig. 4j). These data suggest that uridine supplementation, in turn, may promote regeneration and repair by remodeling metabolic adaptation. We next sought to dissect the cell type-specific effects associated with the regenerative response by constructing a single-nucleus transcriptomic atlas of uridine-treated muscle. We identified 14 muscle cell types, including satellite cells (*Pax7*^+^), the rare muscle stem cell population, fibro-adipogenic progenitors (FAPs, *Pdgfra*^+^), an interstitial mesenchymal cell population that supports muscle regeneration^47^, and fast-twitch muscle fibers (*Mybpc2*^+^ or *Myh1*^+^), that use anaerobic respiration to produce rapid movement bursts (Supplementary Fig. S5a, b). Similar to the bulk RNA-seq results, uridine supplementation restored the expression of genes associated with pyrimidine nucleotide biosynthesis and muscle structure development across cell types, especially in fast-twitch muscle fibers (Supplementary Fig. S5c).

Additionally, the uridine treatment improved the function of the heart underwent myocardial infraction, as evidenced by elevated left ventricular ejection fraction (LVEF) and left ventricular fractional shortening (LVFS) (Fig. 5a, b). Serum lactate dehydrogenase (LDH) and creatine kinase (CK), leakage of which are indicators of acute myocardial infarction, were also lower in uridine-treated mice than control mice (Fig. 5c). Compared to vehicle-treated mice, global gene expression was also reset to be close to the state before injury in uridine-treated mice, with increased expression of genes related to “heart contraction”, and “cardiac muscle tissue development”, and decreased expression of genes associated with “inflammatory response” (Fig. 5d–f). Altogether, uridine promotes the course of tissue regeneration probably by modulating the metabolic process and suppressing inflammation.

**Fig. 6:**
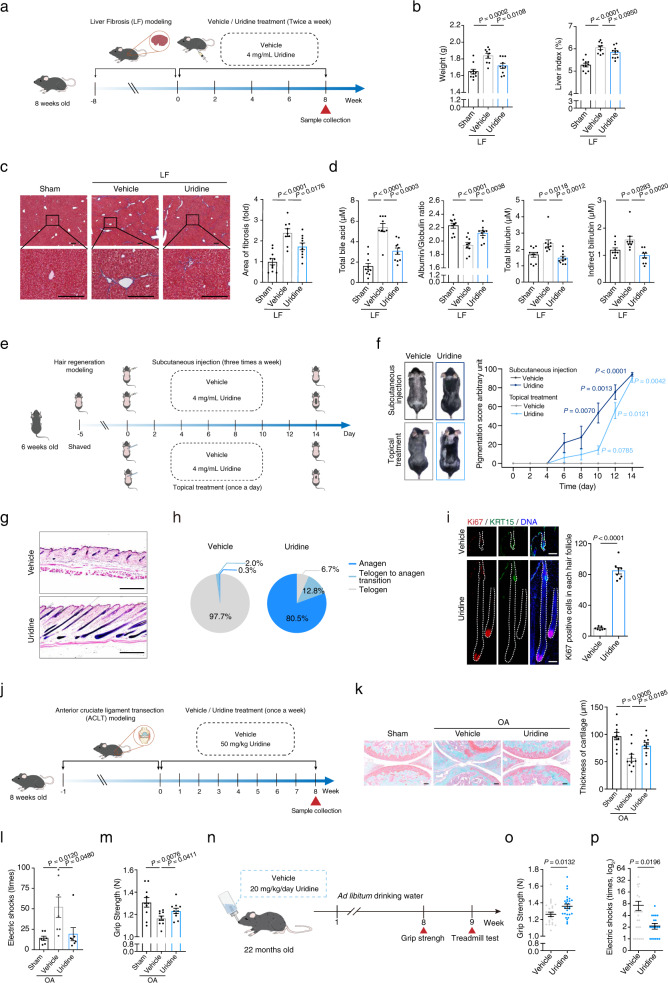
Uridine treatment enhances in vivo tissue regeneration and repair. **a** Schematic diagram for the experimental design of the mouse liver fibrosis (LF) modeling and vehicle or uridine treatment. **b** Bar charts of liver weight (left) and liver index (right) of sham mice (*n* = 10 mice) and liver fibrotic mice treated with vehicle (*n* = 9 mice) or uridine (*n* = 10 mice). **c** Representative images of Masson staining of the liver from sham mice (*n* = 10 mice) and liver fibrotic mice treated with vehicle (*n* = 9 mice) or uridine (*n* = 10 mice). Quantitative data of the relative fibrotic area are shown to the right. Scale bars, 200 μm. **d** Diagnostic tests for liver functions of sham mice (*n* = 10 mice) and liver fibrotic mice treated with vehicle (*n* = 9 mice) or uridine (*n* = 10 mice). **e** Schematic diagram for the time course of the mouse hair regeneration experiment. **f** Hair-growth effect of mice topically treated or subcutaneously injected with vehicle or uridine. Hair-growth rates upon vehicle or uridine treatment were verified by pigmentation scoring. Subcutaneous injection (Vehicle, *n* = 7 mice; Uridine, *n* = 8 mice). Topical treatment (Vehicle, *n* = 6 mice; Uridine, *n* = 10 mice). **g** Representative images of H&E staining of the hair follicle of mice subcutaneously injected with vehicle or uridine at day 14 post treatment. Scale bars, 400 μm. **h** Pie plots showing the hair follicle phase of mice subcutaneously injected with vehicle or uridine at day 14 post treatment. Mean values of hair follicle phases for mice subcutaneously injected with vehicle (*n* = 7 mice) or uridine (*n* = 8 mice) are shown. **i** Ki67 and KRT15 staining of the hair follicle of mice subcutaneously injected with vehicle (*n* = 7 mice) or uridine (*n* = 8 mice) at day 14 post treatment. Scale bars, 200 μm. **j** Schematic diagram for the experimental design of anterior cruciate ligament transection (ACLT) mediated osteoarthritis (OA) modeling and vehicle or uridine treatment. **k** Representative images of Safranin O/ Fast Green staining of articular cartilage from the joints of sham mice (*n* = 10 mice) and OA mice treated with vehicle (*n* = 10 mice) or uridine (*n* = 10 mice). Quantitative data of cartilage thickness are shown to the right. Scale bars, 100 μm. **l** Bar chart showing the times of electric shock for sham mice (*n* = 7 mice) and OA mice treated with vehicle (*n* = 7 mice) or uridine (*n* = 7 mice) on the treadmill within 30 min at day 33 post vehicle or uridine treatment. **m** Bar chart showing the grip strength evaluation of the forelimbs and hind limbs of sham mice (*n* = 10 mice) and OA mice treated with vehicle (*n* = 10 mice) or uridine (*n* = 10 mice) at day 33 post vehicle or uridine treatment. **n** Schematic diagram of the long-term oral administration experiment. **o** Bar chart showing the grip strength evaluation of the forelimbs and hind limbs of mice orally administered vehicle (*n* = 26 mice) or uridine (*n* = 26 mice) at day 57. **p** Bar chart showing the times of electric shock for mice orally administered vehicle (*n* = 26 mice) or uridine (*n* = 22 mice) on the treadmill within 30 min at day 63 post treatment. Data in **b**–**d**, **f**, **i**, **k**–**m**, **o**, and **p** are presented as the means ± SEM. (two-tailed unpaired Student’s *t-*test).

In addition to muscular and cardiac injury models, uridine treatment also facilitates the regeneration of the liver after carbon tetrachloride (CCl_4_) induced injury as evidenced by increased liver-to-body weight ratio and decreased liver fibrosis (Fig. 6a–c). Meanwhile, liver function was restored to a physiological level, such as the total bile acid production (Fig. 6d). In the hair regeneration model, we found that uridine supplementation initiated a new wave of hair growth, as revealed by actively cycling hair follicles with high expression of the proliferation marker Ki67 upon uridine supplementation (Fig. 6e–i). In another tissue injury model, uridine treatment facilitated the regeneration of injured cartilage as assessed by safranin O-fast green staining and further ameliorated functional deterioration, as shown by improved grip strength and athletic ability compared to those of the vehicle-treated group (Fig. 6j–m). Finally, we evaluated the effect of uridine supplementation in physiologically aged mice (22 months old) (Fig. 6n–p) and found improved locomotive activities in the mice with oral administration of uridine for 2 months, as indicated by their enhanced grip strength and exercise endurance (Fig. 6o, p). Overall, by combining systematic metabolomics analysis across multiple models with small-molecule screening for regenerative activity, we identified the endogenous small-molecule metabolite uridine as an effective compound that promotes the repair and regeneration of various tissues and organs, which has the potential to extend the healthspan of aged individuals (Supplementary Fig. S5e).

## Discussion

The fundamental questions we try to answer in this study are how high regenerative capacity is fueled by metabolic mechanisms and how we can enhance regeneration through metabolic intervention. By combining metabolomics and transcriptomics approaches to survey phenotypes that are selectively present in actively regenerating tissues and stem cells across species, we shed new light on cross-species and cross-ages metabolic mechanisms associated with regenerative capacity.

In most vertebrates, tissue regeneration is impaired by aging, due to concomitant with cellular senescence, organ degeneration, and other age-associated comorbidities^2^. The youth factors frequently decreased with age^48,49^, such as polyamines (spermidine or spermine), have been reported to promote the regeneration of tissues and delay the progression of aging-related disorders^50–53^. Metabolic profiling of young tissues and stem cell models with higher regenerative capacity enables us to discover new youth factors and mechanisms associated with regeneration enhancement conserved across species.

The assembly of our metabolomic atlas allowed us to discover metabolic differences between samples with high and low regenerative abilities, but also enabled the identification of dozens of metabolite effectors with the potential to promote tissue regeneration. Here, through the cross-species metabolomics analysis and metabolites screening, we identified endogenous metabolite uridine as a potent regeneration promoting factor. Uridine was identified to be more abundant in tissues or cells with higher regenerative potential. In particular, the concentration of uridine decreased in the plasma from aged individuals, suggesting uridine may navigate a delicate balance between aging and regeneration. In addition, uridine supplementation rejuvenated senescent stem cells, promoted the regeneration and repair of multiple mammalian tissues, and improved the fitness of aged mice. Given the beneficial roles of uridine in promoting tissue repair and improving physiological function, these findings may also have broad relevance for healthy aging treatments. Our study also identified that a single-dose intraperitoneal administration of uridine in mice is sufficient to make the blood uridine concentration reach the concentration required to rejuvenate aged human stem cells in vitro (Fig. 3b and Supplementary Fig. S5d), suggesting a possibility that uridine treatment may result in systemic exposure to uridine to enhance regeneration of different types of tissues in vivo (Supplementary Fig. S5e). Even though the pharmacokinetic study was conducted in rodents, these results also provide useful information for the design of future primate-based preclinical studies and clinical trials on uridine. In a mechanistic view, uridine supplementation reduces inflammation in vitro and in vivo. Supporting our finding, local uridine administration alleviated symptoms of inflammatory bowel disease in mice, concomitant with inhibiting NF-κB signaling^54^. As a test for safety, our data showed that long-term uridine treatment via intraperitoneal injection (up to 5 months) or oral administration (up to 7 months) was nontumorigenic. In fact, beneficial roles of uridine metabolism and uracil analogs in cancer treatment have also been reported^55,56^.

In summary, our study reveals previously unknown metabolism-linked regeneration principles across different species, serving as a mineable resource for investigating regenerative pathways and geroprotective metabolic factors with broad translational potential.

## Materials and methods

### Animal housing and tissue sampling

The use of cynomolgus monkeys and mice in this study was approved by the Ethics Review Committee of the Institute of Zoology, Chinese Academy of Sciences. Monkeys originating from Southeast Asia were housed in cages under a 12-h light-dark cycle at the certified Primate Research Center in Beijing (Xieerxin Biology Resource) and a controlled temperature (22 ± 2 °C) with food and water fed *ad libitum*. C57BL/6 J mice purchased from SiPeiFu (Beijing) Biotechnology Co., Ltd were raised at 25 °C in a 12-h light-dark cycle in the animal care facility at the Institute of Zoology, Chinese Academy of Sciences. All animals were confirmed to have no clinical, experimental, or pregnancy histories prior to the experiment. Randomly selected young (4–6 years old, *n* = 8) and old (18–21 years old, *n* = 8) monkeys were fasted overnight and anesthetized before the perfusion with saline and tissue collection. The cynomolgus monkeys were the same ones used in the previous studies^44,57–59^. Each tissue sample from individual young and old NHPs was systematically taken from strictly identical sampled sites. The tissues were rinsed twice with cold PBS (Gibco) and snap-frozen in liquid nitrogen. The WAT from a young female and plasma from two young females were excluded for metabolomic and/or transcriptomic analysis due to insufficient samples.

Handling and surgical procedures for axolotls (*Ambystoma mexicanum*) were performed following ethical regulations for animal research. For all amputations, animals were narcotized in 0.01% benzocaine (Sigma, E1501) and were later transferred to new tanks with clean water to recover from anesthesia. Tissue collecting at the amputation site was performed at day 0 and 11 post amputation^13,21^.

Human plasma samples from the healthy young male (19–25 years old, *n* = 28) and elderly male (75–92 years old, *n* = 21) individuals were collected at Beijing Hospital and First Affiliated Hospital of Kunming Medical University. The use of human plasma in this study was approved by the Ethics Review Committee of Beijing Hospital.

### Cell culture

Human ESC-derived hMSCs and antler stem cells were cultured on gelatin-coated plates in MSC culture medium containing α-MEM medium (Gibco) supplemented with 10% fetal bovine serum (FBS) (Gibco, Cat# 10099-141), 0.1 mM non-essential amino acids (Gibco), 1% penicillin/streptomycin (Gibco) and 1 ng/mL bFGF (Joint Protein Central, Cat# BBI-EXP-002)^16,60–63^.

### Primary hMSC isolation and culture

Isolation and culture of hPMSCs were performed as previously described^64,65^. In brief, gingiva tissues from a 76-year-old individual were cut by scissors in digestive enzymes containing TrypLE™ Express Enzyme (1×) and Dispase IV. Tissues were incubated at 37 °C for 30 min until small pieces disappeared and then neutralized with MSC culture medium described above. The suspensions were then centrifuged at 200 × *g* for 5 min at room temperature and the resulting pellets were resuspended by MSC culture medium and plated on gelatin-coated plates.

For dASC culture medium (dASC-CM) treatment, during the entire experiments, the hPMSCs of dASC-CM treatment group were cultured in a medium containing 50% filtered dASC culture supernatants and 50% fresh MSC culture medium. The hPMSCs in the vehicle group were cultured in MSC culture medium. Cells from each group with three biological replicates were seeded into 6-well plates (15,000 cells per well) and cultured for three passages before clonal expansion ability detection experiments.

### Metabolome analysis

Frozen tissue samples from axolotls, young and old NHPs (brain, heart, liver, skeletal muscle, white adipose tissue, kidney, skin, and plasma), antler stem cells, and hMSCs were sent to the Calibra-Metabolon Joint Laboratory in Hangzhou for nontargeted metabolomics analysis with Metabolon’s standard protocol. Briefly, this protocol combines Metabolon’s solvent extraction method, UPLC-MS/MS methods that utilize ultra-performance liquid chromatography (UPLC) (Waters, ACQUITY) and high-resolution/accurate mass spectrometry with a heated electrospray ionization (HESI-II) source (Thermo Scientific, Q-Exactive) to obtain relative quantities of a broad spectrum of endogenous compounds. In addition, tissue and cell extracts were analyzed by four fractions: reverse-phase ultrahigh performance liquid chromatography-tandem mass spectroscopy (RP/UPLC-MS/MS) with positive ion mode electrospray ionization (ESI) (water and methanol), RP/UPLC-MS/MS with positive ion mode ESI (water, methanol, and acetonitrile), RP/UPLC-MS/MS with negative-ion mode ESI (water and methanol), and HILIC/UPLC-MS/MS with negative ion mode ESI (water, acetonitrile). For biochemical identification, a proprietary in-house library containing analytical characteristics of pure reference compounds analyzed by each of the four methods was used. These characteristics include retention time, molecular weight to charge ratio (m/z), and associated chromatographic data (including MS/MS spectra). The Calibra-Metabolon Joint Laboratory performed quality control and curation processes, metabolite quantification, and data normalization. The identified metabolites marked with ^*^ are compounds that have not been confirmed using reference standards but their identifications are highly reliable from other available information.

### Cell-based metabolite screening

Metabolites selected from the top hits were purchased from Sigma, or Selleck and initially evaluated in WS hMSCs at 4–9 different concentrations based on their known physiological concentrations (blood concentrations under normal conditions) or previously reported concentrations used in cell culture. Product number, solvent, concentration information and screening results for all metabolites are listed in Supplementary Table S2. The WS hMSCs (P6) were seeded into 96-well plates at a density of 3000 cells for each well and were grown overnight to allow attachment. Metabolites at different concentrations in the fresh medium were added the next day and then changed every other day. On the sixth day after the initial drug treatment, cell proliferation was measured using the IncuCyte S3 live-cell imaging system (Essen BioScience, MI USA)^44^. Cell proliferation capacity was evaluated by phase object confluence (percent) and values of wells with uridine treatment (*n* = 6) were averaged and normalized to wells with vehicle treatment (*n* = 6). The metabolites were further ranked by their effects on cell proliferative potentials in WS hMSCs.

### Measurement of uridine concentration in human and mouse plasma

To study the pharmacokinetic characteristics of uridine in mice after intraperitoneal administration (200 μL of 4 mg/mL uridine in 0.9% NaCl), plasma samples were serially collected post uridine treatment (from 1 to 360 min) for uridine concentration detection. Young (19–25 years old, *n* = 28) and old (75–92 years old, *n* = 21) human plasma samples were collected and used for uridine concentration detection. For sample pretreatment procedure, a 200 μL aliquot of the internal standard (1 μg/mL Fluorouracil (Alta, Cat #1ST10360) in acetonitrile (Sigma, Cat #34851), with 0.1% Formic Acid (Fisher Scientific, Cat #A117-50)) was added to 50 μL of plasma, vortex-mixed for 10 s, and spun in a centrifuge at 15,000 rpm at 4 °C for 10 min. 40 μL of supernatant was diluted with 160 μL water and 10 μL was injected into SCIEX Triple Quad^TM^ 4500 LC-MS/MS System for analysis. For calibrators preparation, uridine (Sigma, Cat# U3003) was dissolved in 50% methanol/H_2_O to get a stock solution (1 mg/mL). The stock solution was further diluted by 50% methanol/H_2_O for calibration curves, which were stocked at –80 °C. Calibrators were diluted to the following concentrations: 0.2, 0.5, 1, 2, 5, 10, 20, 50, 100 μg/mL. The compounds were separated on a reversed-phase column (Kinetex 2.6 μm F5, 100 × 3.0 mm, Phenomenex, Torrance, CA, USA) with the mobile phase. The column was heated to 40 °C, and the mobile phase was eluted at 0.6 mL/min using a Sciex DX Pump. The turbo ion spray interface operated in the negative ion mode at 5500 V and 450 °C. Uridine and fluorouracil (IS) produced mainly deprotonated molecules at m/z 242.6 and 128.9, respectively. The productions were scanned in Q3 after collision with nitrogen in Q2 at m/z 109.0 for uridine and 41.9 for the IS, respectively. Analyst software (version1.6.3, Applied Biosystems) was used for data collection and MultiQuant^TM^ MD Software (version 3.0.2, Applied Biosystems) was used for quantification. Each sample was technically repeated for 6 times, and the mean value was taken for statistical analysis.

### In vitro uridine treatment assay

For hMSC uridine treatment, during the entire experiments, WS hMSCs (passage, P5), HGPS hMSCs (P10) and hPMSCs (P13) of the uridine treatment group were cultured in hMSC culture medium supplemented with 200, 100 and 100 μM uridine (Sigma, U3003), respectively. The cells of the vehicle group were cultured in hMSC culture medium. Each group of cells with three biological replicates were seeded into 6-well plates (30,000 cells for HGPS and WS hMSCs and 15,000 cells for hPMSCs per well) and cultured. Immunostaining of Ki67, H3K9me3, γH2A.X and 53BP1, cell cycle assay and RT-qPCR analysis of repetitive element transcripts in vehicle- or uridine-treated WS hMSCs, HGPS hMSCs and hPMSCs were conducted after two passages with vehicle or uridine treatment.

### RT-qPCR

Total RNA from cells or tissues was extracted by TRIzol (15596018, Gibco) and reverse-transcribed into cDNA using GoScript Reverse Transcription System (A5001, Promega). RT-qPCR was then performed using SYBR qPCR mix (QPS-201, TOYOBO) on a CFX384-Real-time system (Bio-Rad). Primers used for RT-qPCR are listed in the Supplementary Table S3.

### Western blotting assay

Cells were lysed in buffer containing 4% SDS (0227, AMERSCO) followed by BCA quantification of protein concentrations (BCA-02, Beijing Dingguo Changsheng biotechnology Co. Ltd). Proteins (20 μg per sample) were then separated by SDS-PAGE and electrotransferred to PVDF membranes (Millipore). Membranes were blocked in 5% milk, followed by incubation with primary antibodies and horseradish peroxidase-conjugated secondary antibodies. The ChemiDoc XRS^+^ system (Bio-Rad) was used for band visualization and the ImageJ software (NIH) was used for protein quantification analysis of protein levels^66^. Antibodies for western blotting analysis used in this study are listed in Supplementary Table S4.

### Immunofluorescence staining

For tissue samples, tissues were embedded in optimal cutting temperature (OCT) compound, snap-frozen in liquid nitrogen and then stored at –80 °C. Frozen samples were then sectioned at a thickness of 16 μm for further operations. For cell staining, cells were seeded on a coverslip and cultured until 70% confluency. Cells were then fixed (4% formaldehyde for 30 min), permeabilized (0.4% Triton X-100 for 30 min) and blocked (10% donkey serum for 1 h) at room temperature. Next, cells or tissue sections were incubated with indicated primary antibodies at 4 °C overnight followed by secondary antibodies for 1 h at room temperature. A Leica SP5 confocal microscopy was used for imaging, and the ImageJ software (NIH) was used for statistical analysis of fluorescence signals (number, intensity, area as appropriate)^66^. For cell staining, over 100 cells for each biological replicate were quantified. Antibodies used in this study are listed in Supplementary Table S4.

### Flow cytometry analysis

For cell cycle analysis, 5 × 10^5^ cells from each group with three biological replicates were fixed by 70% ethanol at –20 °C overnight and incubated in PBS containing 0.1% Triton X-100, 0.02 mg/mL propidium iodide (P3566, Molecular Probes) and 0.2 mg/mL RNase A (B100675-0500, Sangon biotech) at 37 °C for 30 min. Cells were then analyzed by FACS (BD FACS Calibur).

### Chondrogenesis assay

The detection of chondrogenesis potential was performed as previously described^43,65^. Briefly, after 21 days differentiation, the chondrocytes derived from vehicle- and uridine-treated hMSCs were verified by histochemical staining with toluidine blue (Sigma, T3260).

### SA-β-gal staining

SA-β-gal staining was performed as previously described^25,62,64,67^. Briefly, cells from each group with three biological replicates were fixed (2% formaldehyde and 0.2% glutaraldehyde in PBS) for 5 min at room temperature followed by PBS washing. The cells were then incubated with the staining solution at 37 °C overnight. The quantification of SA-β-gal-positive cells was performed using the ImageJ software (NIH).

### Clonal expansion assay

As previously reported^43,62,64,68^, cells from each group with three biological replicates were seeded into 12-well plates (3,000 cells per well) and cultured for 9–12 days. Then, cells were washed with PBS, fixed with 4% PFA for 30 min, and stained with 0.2% crystal violet for 1 h at room temperature. Cell numbers were quantified using the ImageJ software (NIH).

### Skeletal muscle injury (SMI) assays in mice

Skeletal muscle injury assays were performed as described previously^69^. Briefly, C57BL/6J male mice (8 weeks old) were randomly divided into an uninjured group treated with PBS (Sham group), the injured group treated with PBS or uridine (Injury-vehicle or Injury-uridine group). For muscle injury surgery, mice were firstly anesthetized by 2% isoflurane. And, the skin of the hind legs was disinfected with iodophor. Then a 1.5-cm-long incision was made through the skin overlying the quadriceps femoris muscle. The injury was induced by applying a metal rod pre-cooled with liquid nitrogen to the quadriceps femoris muscle for 5 s. The skin incision was then closed with suture. The injured mice were intraperitoneally injected with 200 μL 0.9% NaCl or 4 mg/mL uridine in 0.9% NaCl every other day from the next day after cryoinjury. Physical functional assays were performed on the day 7 after injury and the mice were sacrificed and sampled on the day 8 after injury.

### Myocardial infarction (MI) assays in mice

Myocardial infarction was induced as previously described^70,71^. First, male C57BL/6 J mice (8 weeks old) were anesthetized with 2% isoflurane in an inducing chamber and immobilized on the surgical board with medical tapes. Next, a 1-cm incision was made on the skin of the left chest and pectoralis major and minor muscles were tore apart. A small hole was created in the fourth intercostal space by a mosquito clamp to allow the heart to pop out. The left anterior descending coronary artery was immediately ligated with a 6-0 silk suture. Right after the ligation, the heart was placed back into the intrathoracic space, followed by air evacuation and closure of the skin incision. For the sham group, mice underwent the same surgical procedures except for the left anterior descending coronary artery ligation and were used as control. Mice with MI surgeries were randomly divided into vehicle and uridine treatment group and received intraperitoneal injection of 200 μL of 0.9% NaCl or 4 mg/mL uridine in 0.9% NaCl on the day of surgery, and supplemented with the same dose every other day from the second to the seventh day.

After 7 days of MI surgery, cardiac function was evaluated through transthoracic echocardiography by using Vevo 2100 imaging system (Visual Sonics, Inc.) with a 30-MHz transducer. Mice were anesthetized with 2% isoflurane. Two-dimensional M-mode traces were obtained at the level of the papillary muscle. Left ventricular ejection fraction (LVEF) and fractional shortening (LVFS) were measured and calculated on three consecutive cardiac cycles. Creatine Kinase (CK) and lactate dehydrogenase (LDH) were measured by chemical analyses on the next day after the surgery. Blood was collected from the fundus venous plexus. Serum was separated through centrifugation at 1000× *g* for 10 min at 4 °C and frozen at –80 °C until use.

### Assay for hair growth in mice

For hair regeneration experiments, C57BL/6J male mice were shaved at postnatal day 43. After five days of observation to ensure that there is no difference in the shaved skin, the mice were treated with uridine or vehicle. Topical administration of uridine (4 mg/mL, solvent formulation: glycerin/water = 8:2) or vehicle (glycerin/water = 8:2) was performed once a day (vehicle control: *n* = 6, uridine: *n* = 10), and subcutaneous injection of uridine (4 mg/mL in PBS) or vehicle (PBS) was performed three times a week (vehicle control: *n* = 7, uridine: *n* = 8). The appearance of skin pigmentation and hair growth was monitored and documented by photos, with the experimenter(s) being blind to the treatment conditions^72^. Progression was also assigned a value from 0 to 100 based on pigmentation levels and hair shaft density, with 0 indicating no hair growth (and no pigmentation) and higher number corresponding to darker skin and larger areas of dense hair growth. Scoring was done blindly. The hair follicle cycling assay was conducted according to a previously reported guideline^73^.

### Assay for liver fibrosis (LF) in mice

Liver fibrosis induction was conducted as previously described^74,75^. Male C57BL/6J mice (8 weeks old) were randomly divided into three groups as follows (*n* = 9–10 per group): sham mice, LF mice treated with vehicle or uridine. Mice of vehicle or uridine groups were intraperitoneally injected with 200 μL CCl_4_ (1 mg/kg) (Sigma, 488488) dissolved in olive oil twice a week for eight weeks to induce liver fibrosis, while the mice in sham group were treated with same dose of 100% olive oil. On the following day, mice were then treated with 200 μL vehicle or 4 mg/mL uridine in 0.9% NaCl by intraperitoneal injection twice a week for eight weeks. Blood samples were collected by eyeball extraction 24 h after the last injection. Serum was separated through centrifugation at 1000× *g* for 10 min at 4 °C. The contents of total bile acid, Albumin/Globulin (A/G) ratio, total bilirubin and indirect bilirubin in serum were analyzed by an automatic biochemical analyzer (TOSHIBA, TBA-120FR). The livers of each group of mice were collected and fixed by 4% paraformaldehyde after perfusion with normal saline for histochemical staining.

### ACLT induced OA assay in mice

ACLT surgery was performed to induce OA as described previously^76–78^. Male C57BL/6J mice (8 weeks old) were randomly divided into three groups (*n* = 10 per group) as follows: sham mice, OA mice treated with vehicle or uridine. For the ACLT surgery, the anterior cruciate ligament of mice in OA groups were transected with microscissors after opening the joint capsule. Seven days after the ACLT surgery, OA mice were injected with 10 μL vehicle (0.9% NaCl) or uridine (50 mg/kg uridine in 0.9% NaCl) into the articular cavity once a week. After 2 months of treatment, the joints were collected for safranin O fast green staining. For Safranin O/ Fast Green staining, mouse joints were collected and fixed in 4% PFA for two days, and then decalcified in 5% methanoic acid for 15 days, finally embedded in paraffin. Sections (4.5 μm) were cut from the paraffin blocks, stained with Fast Green FCF (0.02%) and Safranin O (0.1%), and quantified by measuring the thickness of cartilage with the ImageJ software (NIH).

### Long-term vehicle or uridine administration experiment in physiologically aged mice

For long-term oral administration experiments, aged C57BL/6J male mice (22 months old) were treated with uridine (*n* = 26) or vehicle (*n* = 26) per day. Uridine was mixed with 3 mL drinking water and was given to the mouse at 8:00 a.m. with a dose of 20 mg/kg/day. After drinking 3 mL of water containing uridine, the uridine-treated mice were allowed water fed *ad libitum*. For vehicle treatment, mice were allowed water fed *ad libitum*.

### Physical function measurements in mice

#### Grip strength test

A Grip Strength Meter (Panlab Grid Strength Meter, LE902) was used to measure hind limbs (cryoinjury experiment) and four limbs (forelimbs and hind limbs) (OA and long-term administration experiment) grip strength. The mouse was placed on the top of the grip strength meter. As a mouse grasped the grid, the peak pull force was recorded on a digital force transducer. The mouse was pulled along the direction of the grid at a constant rate until the grip strength meter was released by the mouse. This process was repeated for 10 times with 1 min interval between each time. The mean of the values of the trials excluding the maximum and minimum ones were recorded as the grip strength of each mouse.

#### Treadmill test

For the cryoinjury experiment, mice were trained starting at an initial speed at 5 m/min for 2 min and accelerating to 7 m/min for 2 min and then 9 m/min for 1 min. After two days of training, mice were tested with the starting speed at 5 m/min for 2 min and then accelerating to 63 m/min for 58 min with an acceleration of 1 m/min^2^. The treadmill (SANS Bio Instrument, SA101) was placed at an incline of 5° and set with an electrical stimulation (2 mA). When the mice were unable to return to the treadmill and stayed on the electrode for more than 10 s, the distance (m) of the exhaustion was recorded for each mouse.

For long-term oral administration experiment, mice were trained for two consecutive days at the initial speed for 5 m/min for 5 min and then accelerated to a final speed of 30 m/min with an acceleration of 1 m/min^2^ for 25 min. After two days of training, the mice were tested once a day for three consecutive days, and the times of the electrical stimulation within the 30 min was recorded. The average times of the electrical stimulation detected in three days were recorded for statistical analysis.

#### Rotarod test

The Rota Rod system (Yiyan Tech, YLS-4C) was used for training and detection. For long-term oral administration experiment, mice were trained for three consecutive days by placing each mouse in a different channel on the rod at an initial speed of 4 rpm/min and then accelerating to 44 rpm/min with an acceleration of 8 rpm/min^2^ until it dropped three times during training. Detection was performed for three consecutive days after training, and the average time when the mice dropped down was recorded for statistical analysis.

### Hematoxylin and eosin (H&E) staining

As previously reported^44,79^, tissues were dehydrated in a graded series of alcohols, paraffin-embedded, and sectioned at a thickness of 5 μm with a rotary microtome. For H&E staining, sections were deparaffinized in xylene and rehydrated in gradient alcohols (100%, 100%, 95%, 80%, and 70%) and incubated in hematoxylin solution until the desired degree of staining. Sections were then rinsed in running water for removal of excess hematoxylin, differentiated in 1% acid alcohol for 1 s and then rinsed in running water for 1 min. Lastly, sections were stained with eosin to the desired shade of pink, dehydrated in gradient ethanol and xylene, and mounted with cytoseal-60 (Stephens Scientific).

### Masson’s trichrome staining

To compare the fibrosis among muscles from sham mice and freeze injured mice treated with vehicle or uridine, the Masson’s trichrome stain (Solarbio, G1340) was implemented. The paraffin-embedded sections of muscles were deparaffinized by xylene and rehydrated through gradient alcohols (100%, 100%, 95%, 85%, 75%, and 50%), and running tap water. Then the sections were stained followed by the manufacture’s protocol. The sections were then dehydrated with gradient alcohols (50%, 75%, 85%, 95%, 100%, and 100%), then cleared with xylene and covered with cover slides. The images were taken by the section scanner (Leica, CS2).

### ELISA

The levels of serum proinflammatory cytokines in uridine or vehicle-treated freeze injured mice were tested by ELISA (Thermo scientific, BMS6002 for IL-1β, KMC0061 for IL-6, EMIL7 for IL-7, BMS6005 for MCP-1 and BMS607-3 for TNF-α). The experiment was performed according to the manufacturers’ instructions. The results were quantified by the microplate reader (Thermo scientific, MK3).

### Bulk RNA sequencing

Total RNA of axolotl tissues, premature hMSCs, skin tissues of vehicle- or uridine- treated mice and muscle tissues from non-cryoinjury mice and cryoinjured mice treated with vehicle or uridine for sequencing were extracted by TRIzol (Gibco, 15596018). Construction of transcriptome libraries and high-throughput sequencing for each sample were performed by Novogene Bioinformatics Technology Co. Ltd. Briefly, transcriptome libraries were prepared using NEBNext^®^ Ultra™ Directional RNA Library Prep Kit for Illumina (NEB, USA). The resulting libraries were sequenced on an Illumina platform that generated 150-bp paired-end reads by Novogene Bioinformatics Technology Co. Ltd.

### Nuclei isolation and snRNA-seq on the 10× genomics platform

Nuclei isolation was performed as previously described^57,80–82^. In brief, frozen skeletal muscle tissues from the uninjured mice, injured mice with vehicle or uridine treatment (*n* = 5) were pooled separately and grinded into powder with liquid nitrogen. Then the collection of tissue powder was homogenized by a freezing multi-sample tissue grinding system in 1.5 mL homogenization buffer containing 250 mM sucrose, 25 mM KCl, 5 mM MgCl_2_, 10 mM Tris buffer, 1 μM DTT, 1 × protease inhibitor, 0.4 U/μL RnaseIn (Thermo Fisher Scientific), 0.2 U/μL Superasin (Thermo Fisher Scientific), 0.1% Triton X-100, 1 μM propidium iodide (PI), and 10 ng/mL Hoechst 33342 (Thermo Fisher Scientific) in Nuclease-Free water. Samples were filtered through a 40-micron cell strainer (BD Falcon) twice, and centrifuged at 90× *g* for 2 min at 4 °C. The supernatant was collected and centrifuged at 300× *g* for 5 min at 4 °C, the pellet was resuspended in PBS supplemented with 1% BSA, 0.4 U/μL RnaseIn and 0.2 U/μL Superasin, and filtered again through a 40-micron cell strainer (BD Falcon) prior to sorting. Hoechst 33342 and PI-double-positive nuclei were sorted using FACS (BD Influx) and counted with a dual-fluorescence cell counter (Luna-FL^TM^, Logos Biosystems). Single-nucleus capture and RNA-seq library construction were conducted with a 10× Genomics single-cell 3′ system. Approximately 5,000 nuclei were captured for each sample following the standard 10× capture and library preparation protocol (10× Genomics) and then sequenced in a NovaSeq 6000 sequencing system (Illumina, 20012866).

### Metabolome data analysis

Metabolites were classified into 9 super-pathways (Amino Acid, Carbohydrate, Energy, Lipid, Nucleotide, Peptide, Cofactors and Vitamins, Xenobiotics, and Partially Characterized Molecules) and sub-pathways (Polyamine Metabolism, Aminosugar Metabolism, Lysine Metabolism, etc.). Super-pathways and sub-pathways were annotated by Metabolon’s internal database.

For statistical analysis and data display, any missing values were assumed to be below the limit of detection; the raw value of area counts for each biochemical were imputed with the compound minimum and then rescaled to set the median equal to 1 (normalized value). DPMPs analysis was carried out with MetaboAnalyst (version 4.0) based on the normalized value for each metabolite, which was then auto-scaled^39^. DPMPs were identified with a cutoff of *P*-value < 0.05.

The differential abundance score for each super-pathway or sub-pathway was calculated as previously reported^83^. The differential abundance (DA) score was calculated as

$${{{\mathrm{DA}}}}\;{{{\mathrm{score}}}} = \frac{{{{{\mathrm{number}}}}\;{{{\mathrm{of}}}}\;{{{\mathrm{increased}}}}\;{{{\mathrm{metabolites}}}} - {{{\mathrm{number}}}}\;{{{\mathrm{of}}}}\;{{{\mathrm{decreased}}}}\;{{{\mathrm{metabolites}}}}}}{{{{{\mathrm{number}}}}\;{{{\mathrm{of}}}}\;{{{\mathrm{identified}}}}\;{{{\mathrm{metabolites}}}}\;{{{\mathrm{in}}}}\;{{{\mathrm{pathway}}}}}}$$

The DA score ranges from –1 to 1. A DA score of –1 means that all identified metabolites in the super-pathway or sub-pathway was decreased, and a DA score of 1 means that all metabolites in the indicated super-pathway or sub-pathway was increased.

To evaluate metabolome data reproducibility, partial least squares discrimination analysis (PLS-DA) was conducted by MetaboAnalyst (version 4.0) and the results were visualized by R package ggplot2 (version 3.3.2)^39^.

Intra-tissue temporal correlations among metabolites were assessed via a permutation test by performing 100 random permutations of the replicates in each sample and estimating the corresponding correlation coefficient and significance by *Pearson*’s correlation in R (version 4.0.2). Correlation heatmaps were generated based on the mean correlation coefficient of all permutation tests using R pheatmap package (version 1.0.12).

### Bulk RNA-seq data processing

As reported previously^76,84^, pair-end raw reads were first trimmed with the TrimGalore (version 0.4.5) (Babraham Bioinformatics) (https://github.com/FelixKrueger/TrimGalore). For hMSC samples, cynomolgus monkeys and mice tissues, cleaned reads were then mapped to the human (*Homo sapiens*) hg19, or cynomolgus macaque (*Macaca Fascicularis*) MacFas5.0 or mouse (*Mus musculus*) mm10 genome obtained from UCSC genome browser database using hisat2 (version 2.0.4)^85^. For reads alignment of the RNA-seq data of axolotl limb samples, cleaned reads were aligned to the reference genome assembly v3.0.0 (AmexG_v3.0.0, https://www.axolotl-omics.org/assemblies). Read counts for each gene were then calculated by HTSeq (version 0.11.0) and only high-quality mapped reads (score of mapping quality more than 20) were further analyzed^86^. Differentially expressed genes (DEGs) were calculated by R package DESeq2 (version 1.22.2) with a cutoff of Benjamini-Hochberg adjusted *P*-value < 0.05 and |log_2_(fold change)| > 0.25 for axolotl tissues, hMSCs, and mice tissues and *P*-value < 0.05 and |log_2_(fold change)| > 0.25 for NHP tissues^87^. The annotation for axolotl genes was conducted following a previous study^21^. Downregulated genes in injured muscle or heart samples and then restored upon uridine treatment were termed as “rescue DEGs (upregulated)”, and upregulated genes in injured muscle or heart samples and then restored upon uridine treatment were termed as “rescue DEGs (downregulated)”.

### Gene ontology (GO), pathway and gene set analysis

GO and pathway enrichment analyses were conducted using Metascape^88^.

Gene set enrichment analysis was conducted using GSEA (version 4.1.0) with default parameters^89^.

The differential expression (DE) score for the indicated pathway was calculated as

$${\mathrm{DE}}\;{{{\mathrm{score}}}} = \frac{{{{{\mathrm{number}}}}\;{{{\mathrm{of}}}}\;{{{\mathrm{differentially}}}}\;{{{\mathrm{expressed}}}}\;{{{\mathrm{genes}}}}}}{{{{{\mathrm{number}}}}\;{{{\mathrm{of}}}}\;{{{\mathrm{genes}}}}\;{{{\mathrm{in}}}}\;{{{\mathrm{pathway}}}}}}$$

Mitochondria-localized genes were obtained from MitoCarta database (version 3.0)^90^.

Regeneration-associated genes were obtained from REGene database^91^.

Metabolic genes were obtained from Kyoto Encyclopedia of Genes and Genomes (KEGG) database^92^.

### Transcription factor (TF) enrichment analysis

Transcription factor enrichment analysis was performed using R package RcisTarget (version 1.10.0)^93^. The transcription factor networks were visualized with Cytoscape (version 3.8.0)^94^.

### Metabolic pathway enrichment analysis with transcriptomic and metabolomic data

The integrated pathway-level analysis of transcriptomic and metabolomic data was conducted with “Joint Pathway Analysis” module in MetaboAnalyst (version 4.0)^39^.

### Analysis of snRNA-seq data

snRNA-seq data were processed with Cell Ranger (version 3.1.0). The pre-mRNA *Mus musculus* (version mm10) reference was built following the Cell Ranger protocol (https://support.10xgenomics.com/single-cell-gene-expression/software/pipelines/latest/advanced/references).

The expression matrix from Cell Ranger output was calculated with Seurat (version 3.1.3)^95^. Cells with more than 200 genes and with a mitochondrial gene ratio of fewer than 5% were kept. Doublets were identified using DoubletFinder (version 2.0.2)^96^. Upon normalization and clustering as described below, a cluster with relatively higher mitochondrial gene ratios and lower unique molecular identifier (UMI) counts were also excluded. Finally, 22,713 high-quality cells were retained for downstream analyses.

Dataset of each sample was normalized by “SCTransfrom” function of Seurat. After datasets from different samples were integrated and scaled, the principal component analysis was performed with the “RunPCA” function, and clusters were then identified using the “FindClusters” function. Dimensionality reduction was conducted with the “RunUMAP” function. Differential gene expression analysis was performed using the “FindMarkers” function of Seurat between different groups with the Wilcox test. DEGs were identified as those with adjusted *P*-value < 0.05 and |log_2_(fold change)| > 0.25.

### Statistical analysis

Data are presented as the means ± SEM. *P*-value (*P*) was calculated using GraphPad Prism 9 software by two-tailed Student’s *t-*test. Statistical methods are indicated in figure legends.

## Supplementary information

Supplementary Figures S1-S5

Supplementary Table S1

Supplementary Table S2

Supplementary Table S3

Supplementary Table S4

## Data Availability

The raw sequence data of RNA-seq generated in this study have been deposited in the Genome Sequence Archive in National Genomics Data Center under accession numbers CRA002966, CRA002968, CRA002965, CRA003067, CRA003064, CRA004077, and CRA004294. The differentially present metabolic products and differentially expressed genes identified in our regenerative or uridine-treated systems have been deposited to the Regeneration Roadmap (RR, https://ngdc.cncb.ac.cn/aging/index) or Aging Atlas (AA, https://ngdc.cncb.ac.cn/aging/index) databases^97,98^.
