# Supplementary material for: Cross-species metabolomic analysis identifies uridine as a potent regeneration promoting factor
Source: Cell Discov. 2022 Feb 1;8:6. doi: 10.1038/s41421-021-00361-3 (PMC8803930; doi:10.1038/s41421-021-00361-3)
Supplement: Supplementary file 1 — Supplementary Figures S1-S5 [file 41421_2021_361_MOESM1_ESM.pdf]

Supplementary Information

Cross-species metabolomic analysis identifies uridine as a potent regeneration promoting factor

Zunpeng Liu[1,2,3,#], Wei Li[4,5,#], Lingling Geng[4,5,#], Liang Sun[6,7,#], Qiaoran Wang[3,8,9,#], Yang Yu[10,#], Pengze Yan[3,11], Chuqian Liang[3,11], Jie Ren[2,3,8,9], Moshi Song[2,3,11], Qian Zhao[4,5], Jinghui Lei[4,5], Yusheng Cai[2,11,12], Jiaming Li[3,8,9], Kaowen Yan[2,11,12], Zeming Wu[2,11,12], Qun Chu[1,2,12], Jingyi Li[2,11,12], Si Wang[4,5], Chunyi Li[13], Jing-Dong J. Han[14], Reyna Hernandez-Benitez[15], Ng Shyh-Chang[1,2,3], Juan Carlos Izpisua Belmonte[15], Weiqi Zhang[2,3,8,9,*], Jing Qu[1,2,3,12,*], Guang-Hui Liu[2,3,4,5,11,12,*]

[1]State Key Laboratory of Stem Cell and Reproductive Biology, Institute of Zoology, Chinese Academy of Sciences, Beijing, China

[2]Institute for Stem cell and Regeneration, CAS, Beijing, China

[3]University of Chinese Academy of Sciences, Beijing, China

[4]Advanced Innovation Center for Human Brain Protection, National Clinical Research Center for Geriatric Disorders, Xuanwu Hospital Capital Medical University, Beijing, China

[5]Aging Translational Medicine Center, Xuanwu Hospital, Capital Medical University, Beijing, China

[6]The Key Laboratory of Geriatrics, Beijing Institute of Geriatrics, Institute of Geriatric Medicine, Chinese Academy of Medical Sciences, Beijing Hospital/National Center of Gerontology of National Health Commission, Beijing, China

[7]The NHC Key Laboratory of Drug Addiction Medicine, Kunming Medical University, Kunming, China

[8]CAS Key Laboratory of Genomic and Precision Medicine, Beijing Institute of Genomics, Chinese Academy of Sciences, Beijing, China

[9]China National Center for Bioinformation, Beijing, China

[10]Beijing Key Laboratory of Reproductive Endocrinology and Assisted Reproductive Technology and Key Laboratory of Assisted Reproduction, Department of Obstetrics and Gynecology, Ministry of Education, Center for Reproductive Medicine, Peking University Third Hospital, Beijing, China

[11]State Key Laboratory of Membrane Biology, Institute of Zoology, Chinese Academy of Sciences, Beijing, China

[12]Beijing Institute for Stem Cell and Regenerative Medicine, Beijing, China

[13]Institute of Antler Science and Product Technology, Changchun Sci-Tech University, Changchun, China

[14]Peking-Tsinghua Center for Life Sciences, Academy for Advanced Interdisciplinary Studies, Center for Quantitative Biology (CQB), Peking University, Beijing, China

[15]Gene Expression Laboratory, Salk Institute for Biological Studies, La Jolla, CA, USA

[#]These authors contributed equally

[*]Correspondence: zhangwq@big.ac.cn (WZ), qujing@ioz.ac.cn (JQ), ghliu@ioz.ac.cn (GHL)

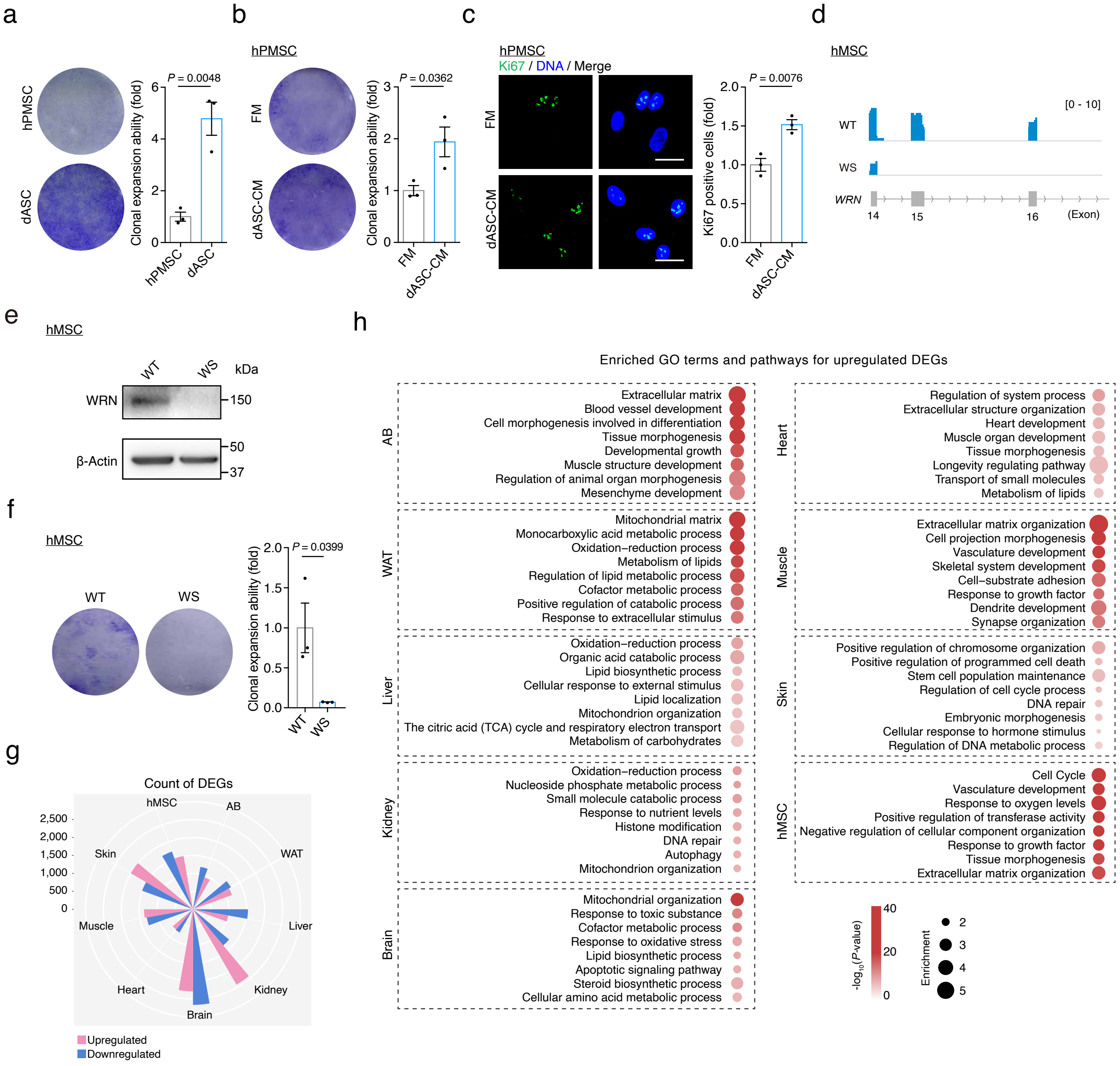
a
hPMSC
dASC
Clonal expansion ability (fold)
P = 0.0048
b
hPMSC
FM
dASC-CM
Clonal expansion ability (fold)
P = 0.0362
c
hPMSC
Ki67 / DNA / Merge
FM
dASC-CM
Ki67 positive cells (fold)
P = 0.0076
d
hMSC
[0 - 10]
WT
WS
WRN
14
15
16
(Exon)
e
hMSC
WT
WS
kDa
WRN
150
β-Actin
50
37
f
hMSC
WT
WS
Clonal expansion ability (fold)
P = 0.0399
g
Count of DEGs
hMSC
AB
WAT
Liver
Kidney
Brain
Heart
Muscle
Skin
2,500
2,000
1,500
1,000
500
0
Upregulated
Downregulated
h
Enriched GO terms and pathways for upregulated DEGs
AB
Extracellular matrix
Blood vessel development
Cell morphogenesis involved in differentiation
Tissue morphogenesis
Developmental growth
Muscle structure development
Regulation of animal organ morphogenesis
Mesenchyme development
WAT
Mitochondrial matrix
Monocarboxylic acid metabolic process
Oxidation–reduction process
Metabolism of lipids
Regulation of lipid metabolic process
Cofactor metabolic process
Positive regulation of catabolic process
Response to extracellular stimulus
Liver
Oxidation–reduction process
Organic acid catabolic process
Lipid biosynthetic process
Cellular response to external stimulus
Lipid localization
Mitochondrion organization
The citric acid (TCA) cycle and respiratory electron transport
Metabolism of carbohydrates
Kidney
Oxidation–reduction process
Nucleoside phosphate metabolic process
Small molecule catabolic process
Response to nutrient levels
Histone modification
DNA repair
Autophagy
Mitochondrion organization
Brain
Mitochondrial organization
Response to toxic substance
Cofactor metabolic process
Response to oxidative stress
Lipid biosynthetic process
Apoptotic signaling pathway
Steroid biosynthetic process
Cellular amino acid metabolic process
Heart
Regulation of system process
Extracellular structure organization
Heart development
Muscle organ development
Tissue morphogenesis
Longevity regulating pathway
Transport of small molecules
Metabolism of lipids
Muscle
Extracellular matrix organization
Cell projection morphogenesis
Vasculature development
Skeletal system development
Cell–substrate adhesion
Response to growth factor
Dendrite development
Synapse organization
Skin
Positive regulation of chromosome organization
Positive regulation of programmed cell death
Stem cell population maintenance
Regulation of cell cycle process
DNA repair
Embryonic morphogenesis
Cellular response to hormone stimulus
Regulation of DNA metabolic process
hMSC
Cell Cycle
Vasculature development
Response to oxygen levels
Positive regulation of transferase activity
Negative regulation of cellular component organization
Response to growth factor
Tissue morphogenesis
Extracellular matrix organization
-log10(P-value)
40
20
0
Enrichment
2
3
4
5

Supplementary Fig. S1. Cross-species transcriptomic profiles underlying differential regenerative capacities, Related to Fig.1.

a Clonal expansion analysis of hPMSCs and dASCs. Quantitative data to the right are presented as the mean ± SEM (Two-tailed unpaired Student's *t* test). *n* = 3 biological replicates. **b** Clonal expansion analysis of hPMSCs (passage 13, P13) cultured in fresh medium (FM) and dASC culture medium (dASC-CM) for three passages. Quantitative data to the right are presented as the mean ± SEM (Two-tailed unpaired Student's *t* test). *n* = 3 biological replicates. **c** Ki67 staining of hPMSCs (P13) cultured in FM and dASC-CM for three passages. Quantitative data to the right are presented as the mean ± SEM (Two-tailed unpaired Student's *t* test). *n* = 3 biological replicates. Scale bars, 25 μm. **d** Representative tracks of RNA-seq data showing the RNA levels of *WRN* in WT and WS hMSCs (P9). **e** Western blot analysis of WRN levels in WT and WS hMSCs (P9). β-Actin was used as the loading control. **f** Clonal expansion analysis of WT and WS hMSCs (P9). Quantitative data to the right are presented as the mean ± SEM (Two-tailed unpaired Student's *t* test). *n* = 3 biological replicates. **g** Wind-rose plot showing the count of DEGs in AB at DPA 11, young NHP tissues, and young hMSCs. **h** Bubble plot showing the enriched GO terms and pathways for upregulated DEGs in AB at DPA 11, young NHP tissues, and young hMSCs. The color key from white to red indicates low to high $-\log_{10}$(*P*-value). The bubble sizes are positively correlated to the relative enrichment levels.

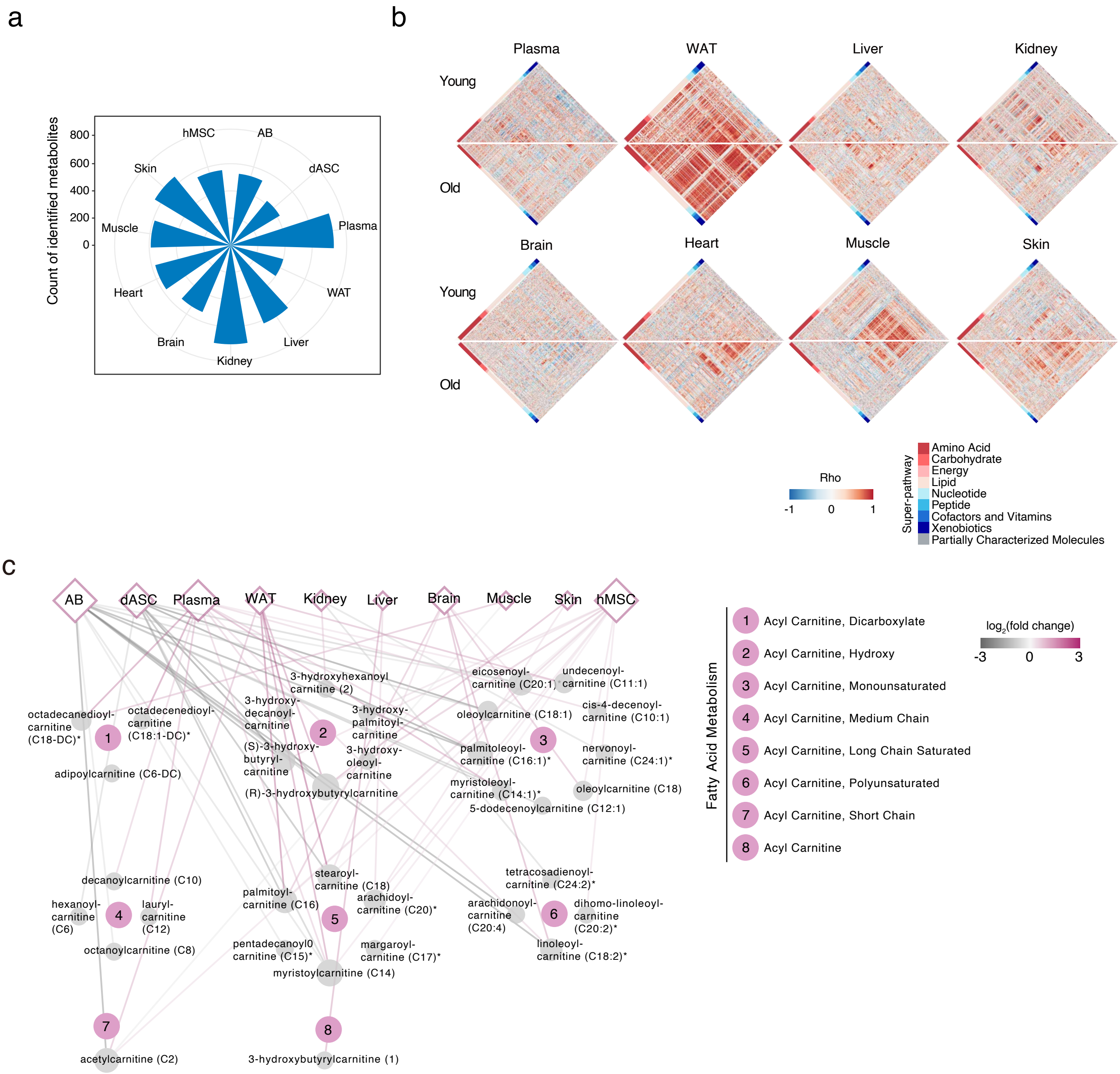
a
Count of identified metabolites
800
600
400
200
0
hMSC
AB
dASC
Skin
Plasma
Muscle
Heart
WAT
Brain
Liver
Kidney
b
Plasma
WAT
Liver
Kidney
Young
Old
Brain
Heart
Muscle
Skin
Young
Old
Rho
-1
0
1
Super-pathway
Amino Acid
Carbohydrate
Energy
Lipid
Nucleotide
Peptide
Cofactors and Vitamins
Xenobiotics
Partially Characterized Molecules
c
AB
dASC
Plasma
WAT
Kidney
Liver
Brain
Muscle
Skin
hMSC
3-hydroxyhexanoyl carnitine (2)
eicosenoyl-carnitine (C20:1)
undecenoyl-carnitine (C11:1)
octadecanedioyl-carnitine (C18-DC)*
octadecenedioyl-carnitine (C18:1-DC)*
3-hydroxy-decanoyl-carnitine
3-hydroxy-palmitoyl-carnitine
oleoylcarnitine (C18:1)
cis-4-decenoyl-carnitine (C10:1)
(S)-3-hydroxy-butyryl-carnitine
3-hydroxy-oleoyl-carnitine
palmitoleoyl-carnitine (C16:1)*
nervonoyl-carnitine (C24:1)*
adipoylcarnitine (C6-DC)
(R)-3-hydroxybutyrylcarnitine
myristoleoyl-carnitine (C14:1)*
oleoylcarnitine (C18)
5-dodecenoylcarnitine (C12:1)
decanoylcarnitine (C10)
stearoyl-carnitine (C18)
tetracosadienoyl-carnitine (C24:2)*
palmitoyl-carnitine (C16)
arachidoyl-carnitine (C20)*
arachidonoyl-carnitine (C20:4)
dihomo-linoleoyl-carnitine (C20:2)*
hexanoyl-carnitine (C6)
lauryl-carnitine (C12)
octanoylcarnitine (C8)
pentadecanoyl0 carnitine (C15)*
margaroyl-carnitine (C17)*
linoleoyl-carnitine (C18:2)*
myristoylcarnitine (C14)
acetylcarnitine (C2)
3-hydroxybutyrylcarnitine (1)
Fatty Acid Metabolism
1 Acyl Carnitine, Dicarboxylate
2 Acyl Carnitine, Hydroxy
3 Acyl Carnitine, Monounsaturated
4 Acyl Carnitine, Medium Chain
5 Acyl Carnitine, Long Chain Saturated
6 Acyl Carnitine, Polyunsaturated
7 Acyl Carnitine, Short Chain
8 Acyl Carnitine
log2(fold change)
-3
0
3

Supplementary Fig. S2. Metabolomic profiles for cross-species models with differential regenerative abilities, Related to Fig.2.

a Wind-rose plots showing the count of identified metabolites in AB, dASCs, NHP tissues, and hMSCs. **b** Intra-tissue correlation heatmaps for metabolites in each tissue from young and old NHPs. The correlation coefficient rho from negative to positive is shown from blue to red. **c** Network diagram showing the representative sub-pathways associated with "Fatty Acid Metabolism (Acyl Carnitine)" and relative abundance for DPMPs for each sub-pathway in AB at DPA 11, dASCs, young NHP tissues, and young hMSCs. The color of the edge from black to amaranth indicates log_2(fold change) from low to high. The node sizes are positively correlated to the edge counts for each node.

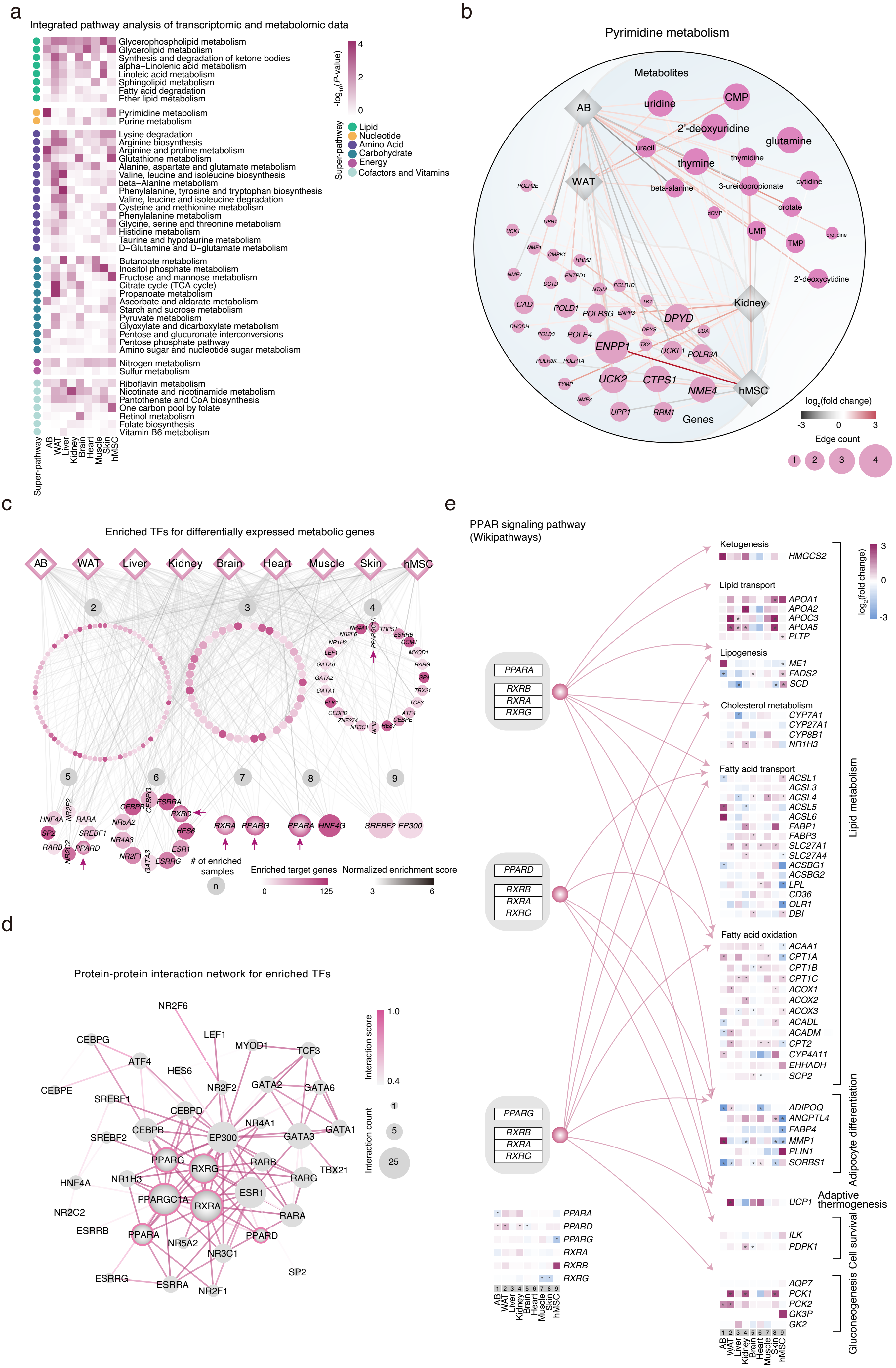
a
Integrated pathway analysis of transcriptomic and metabolomic data
Glycerophospholipid metabolism
Glycerolipid metabolism
Synthesis and degradation of ketone bodies
alpha–Linolenic acid metabolism
Linoleic acid metabolism
Sphingolipid metabolism
Fatty acid degradation
Ether lipid metabolism
Pyrimidine metabolism
Purine metabolism
Lysine degradation
Arginine biosynthesis
Arginine and proline metabolism
Glutathione metabolism
Alanine, aspartate and glutamate metabolism
Valine, leucine and isoleucine biosynthesis
beta–Alanine metabolism
Phenylalanine, tyrosine and tryptophan biosynthesis
Valine, leucine and isoleucine degradation
Cysteine and methionine metabolism
Phenylalanine metabolism
Glycine, serine and threonine metabolism
Histidine metabolism
Taurine and hypotaurine metabolism
D–Glutamine and D–glutamate metabolism
Butanoate metabolism
Inositol phosphate metabolism
Fructose and mannose metabolism
Citrate cycle (TCA cycle)
Propanoate metabolism
Ascorbate and aldarate metabolism
Starch and sucrose metabolism
Pyruvate metabolism
Glyoxylate and dicarboxylate metabolism
Pentose and glucuronate interconversions
Pentose phosphate pathway
Amino sugar and nucleotide sugar metabolism
Nitrogen metabolism
Sulfur metabolism
Riboflavin metabolism
Nicotinate and nicotinamide metabolism
Pantothenate and CoA biosynthesis
One carbon pool by folate
Retinol metabolism
Folate biosynthesis
Vitamin B6 metabolism
Super-pathway
AB
WAT
Liver
Kidney
Brain
Heart
Muscle
Skin
hMSC
-log10(P-value)
4
2
0
Super-pathway
Lipid
Nucleotide
Amino Acid
Carbohydrate
Energy
Cofactors and Vitamins
b
Pyrimidine metabolism
Metabolites
AB
WAT
Kidney
hMSC
uridine
CMP
2'-deoxyuridine
glutamine
uracil
thymine
thymidine
beta-alanine
3-ureidopropionate
cytidine
orotate
UMP
TMP
orotidine
2'-deoxycytidine
dCMP
CAD
POLD1
POLR3G
DPYD
POLE4
ENPP1
UCKL1
POLR3A
UCK2
CTPS1
NME4
UPP1
RRM1
Genes
log2(fold change)
-3
0
3
Edge count
1
2
3
4
c
Enriched TFs for differentially expressed metabolic genes
AB
WAT
Liver
Kidney
Brain
Heart
Muscle
Skin
hMSC
2
3
4
5
6
7
8
9
HNF4A
NR2F2
RARA
SP2
SREBF1
RARB
PPARD
NR2C2
CEBPB
CEBPG
ESRRA
RXRG
NR5A2
HES6
NR4A3
ESR1
NR2F1
GATA3
ESRRG
RXRA
PPARG
PPARA
HNF4G
SREBF2
EP300
# of enriched samples
n
Enriched target genes
0
125
Normalized enrichment score
3
6
d
Protein-protein interaction network for enriched TFs
NR2F6
CEBPG
LEF1
MYOD1
TCF3
ATF4
HES6
CEBPE
NR2F2
GATA2
GATA6
SREBF1
CEBPD
NR4A1
CEBPB
EP300
GATA3
GATA1
SREBF2
PPARG
RXRG
RARB
TBX21
HNF4A
NR1H3
RARG
ESR1
PPARGC1A
RXRA
NR2C2
RARA
ESRRB
PPARA
NR5A2
PPARD
NR3C1
SP2
ESRRG
ESRRA
NR2F1
Interaction score
1.0
0.4
Interaction count
1
5
25
e
PPAR signaling pathway
(Wikipathways)
PPARA
RXRB
RXRA
RXRG
PPARD
RXRB
RXRA
RXRG
PPARG
RXRB
RXRA
RXRG
Ketogenesis
HMGCS2
Lipid transport
APOA1
APOA2
APOC3
APOA5
PLTP
Lipogenesis
ME1
FADS2
SCD
Cholesterol metabolism
CYP7A1
CYP27A1
CYP8B1
NR1H3
Fatty acid transport
ACSL1
ACSL3
ACSL4
ACSL5
ACSL6
FABP1
FABP3
SLC27A1
SLC27A4
ACSBG1
ACSBG2
LPL
CD36
OLR1
DBI
Fatty acid oxidation
ACAA1
CPT1A
CPT1B
CPT1C
ACOX1
ACOX2
ACOX3
ACADL
ACADM
CPT2
CYP4A11
EHHADH
SCP2
Lipid metabolism
ADIPOQ
ANGPTL4
FABP4
MMP1
PLIN1
SORBS1
Adipocyte differentiation
UCP1
Adaptive thermogenesis
ILK
PDPK1
Cell survival
AQP7
PCK1
PCK2
GK3P
GK2
Gluconeogenesis
log2(fold change)
3
0
-3
PPARA
PPARD
PPARG
RXRA
RXRB
RXRG
1 2 3 4 5 6 7 8 9
AB
WAT
Liver
Kidney
Brain
Heart
Muscle
Skin
hMSC

Supplementary Fig. S3. Transcriptional regulation of metabolic pathways associated with regenerative capacities, Related to Fig.2.

a Heatmap showing the enriched metabolic pathways based on the interplay analysis of transcriptome and metabolome data. Integrated pathway analysis of transcriptomic and metabolomic data were conducted by the MetaboAnalyst web tool based on the upregulated DEGs and increased DPMPs in samples with higher regenerative potential. The color key from white to amaranth indicates $-\log_{10}(P\text{-value})$ from low to high. **b** Network diagram showing the differential levels for DEMGs and DPMPs in pyrimidine metabolism sub-pathway in samples with higher regenerative potential. The color of the edge from black to amaranth indicates $\log_2$(fold change) from low to high. The node sizes are positively correlated to the edge counts for each node. The DEMGs and DPMPs of pyrimidine metabolism enriched in AB at DPA 11, young WAT, kidney and young hMSCs are shown. **c** Network diagram showing the enriched transcription factors (TFs) for DEMGs in samples with higher regenerative potential. The color of the edge from white to black indicates enrichment level (Normalized enrichment level, NES) for TF from low to high. The color of the node from white to amaranth indicates count of enriched target genes for the corresponding TF. "# of enriched samples (n)" means the count of enriched models for indicated TF. TFs enriched in at least two types of samples with higher regenerative potential are shown. **d** Protein-protein interaction network showing the interaction of the enriched TFs for DEMGs in samples with higher regenerative potential. TFs enriched in at least four types of samples with higher regenerative potential were retained for protein-protein interaction analysis. The color of the edge from white to amaranth indicates the interaction score from low to high. Protein-protein interaction was calculated by STRING database (https://cn.string-db.org) and visualized by Cytoscape. **e** Network diagrams and heatmaps showing the relative expression levels for genes in PPAR signaling pathway. The color key of the heatmap from blue to amaranth indicates the relative expression levels [$\log_2$(fold change)] of indicated genes from low to high. Heatmap cells marked by * indicate significantly changed DEGs.

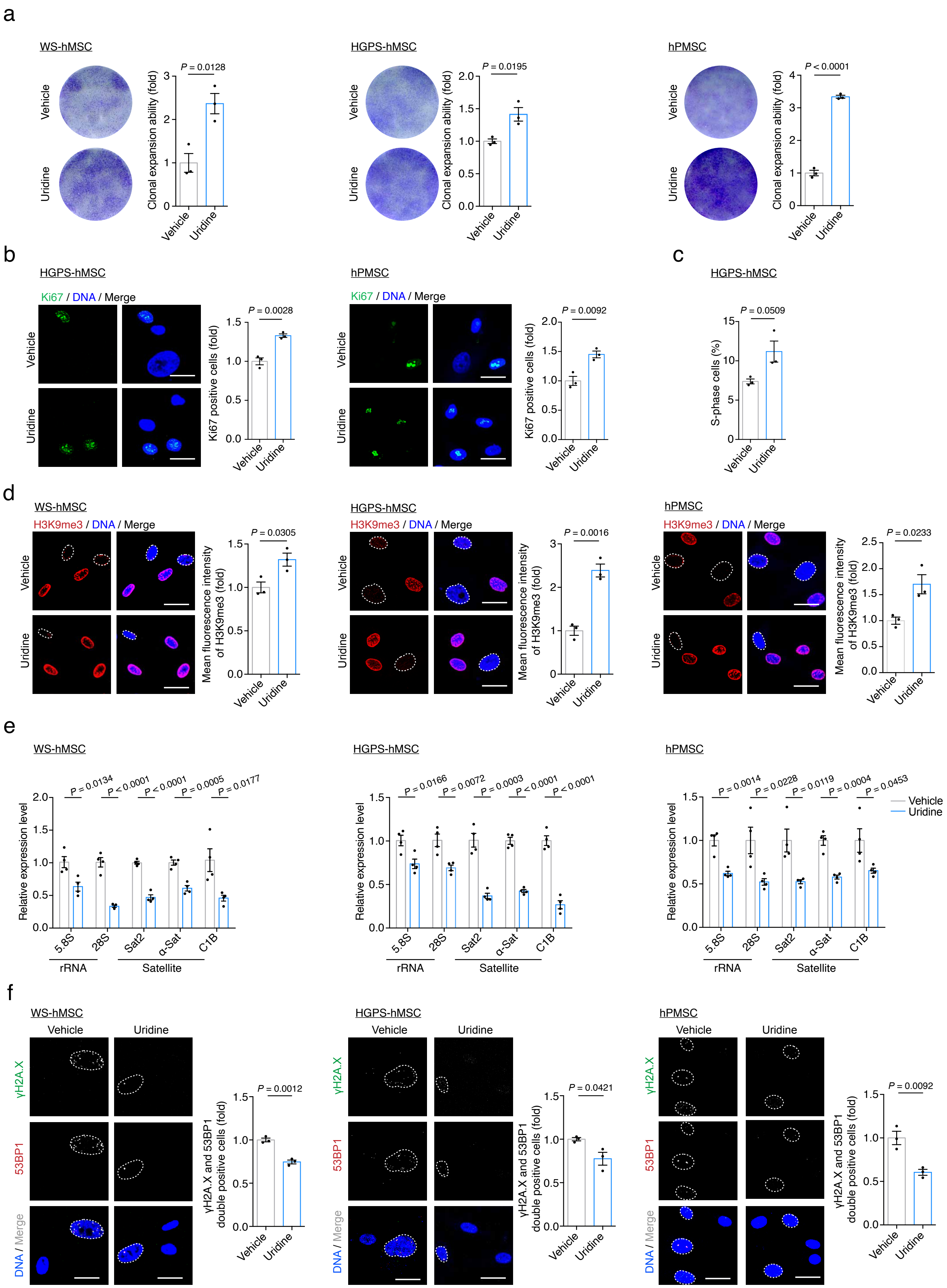

a
WS-hMSC
HGPS-hMSC
hPMSC
Vehicle
Uridine
Clonal expansion ability (fold)
P = 0.0128
P = 0.0195
P < 0.0001
b
HGPS-hMSC
hPMSC
Ki67 / DNA / Merge
Ki67 positive cells (fold)
P = 0.0028
P = 0.0092
c
HGPS-hMSC
S-phase cells (%)
P = 0.0509
d
WS-hMSC
HGPS-hMSC
hPMSC
H3K9me3 / DNA / Merge
Mean fluorescence intensity of H3K9me3 (fold)
P = 0.0305
P = 0.0016
P = 0.0233
e
WS-hMSC
HGPS-hMSC
hPMSC
Relative expression level
P = 0.0134
P < 0.0001
P < 0.0001
P = 0.0005
P = 0.0177
P = 0.0166
P = 0.0072
P = 0.0003
P < 0.0001
P < 0.0001
P = 0.0014
P = 0.0228
P = 0.0119
P = 0.0004
P = 0.0453
5.8S
28S
Sat2
α-Sat
C1B
rRNA
Satellite
Vehicle
Uridine
f
WS-hMSC
HGPS-hMSC
hPMSC
γH2A.X
53BP1
DNA / Merge
γH2A.X and 53BP1 double positive cells (fold)
P = 0.0012
P = 0.0421
P = 0.0092

Supplementary Fig. S4. Uridine treatment enhances hMSC activity, Related to Fig.3.

a Left, clonal expansion analysis of vehicle- and uridine (200 μM)-treated WS hMSCs (P5) at P2 post treatment. Middle, clonal expansion analysis of vehicle- and uridine (100 μM)-treated HGPS hMSCs (P10) at P2 post treatment. Right, clonal expansion analysis of vehicle- and uridine (100 μM)-treated hPMSCs (P13) at P2 post treatment. Data are presented as the mean ± SEM (Two-tailed unpaired Student's *t* test). *n* = 3 biological replicates. **b** Left, immunostaining of Ki67 in vehicle- and uridine (100 μM)-treated HGPS hMSCs (P10) at P2 post treatment. Right, immunostaining of Ki67 in vehicle- and uridine (100 μM)-treated hPMSCs (P13) at P2 post treatment. Data are presented as the mean ± SEM (Two-tailed unpaired Student's *t* test). *n* = 3 biological replicates. Scale bars, 25 μm. **c** Cell cycle analysis of vehicle- and uridine (100 μM)-treated HGPS hMSCs (P10) at P2 post treatment. Data are presented as the mean ± SEM (Two-tailed unpaired Student's *t* test). *n* = 3 biological replicates. **d** Left, immunostaining of H3K9me3 in vehicle- and uridine (200 μM)-treated WS hMSCs (P5) at P2 post treatment. Middle, immunostaining of H3K9me3 in vehicle- and uridine (100 μM)-treated HGPS hMSCs (P10) at P2 post treatment. Right, immunostaining of H3K9me3 in vehicle- and uridine (100 μM)-treated hPMSCs (P13) at P2 post treatment. Dashed lines indicate the nuclear boundaries of the cells with decreased H3K9me3 signals. Data are presented as the mean ± SEM (Two-tailed unpaired Student's *t* test). *n* = 3 biological replicates. Scale bars, 25 μm. **e** Left, RT-qPCR analysis of transcripts from indicated repetitive elements in vehicle- and uridine (200 μM)-treated WS hMSCs (P5) at P2 post treatment. Middle, RT-qPCR analysis of transcripts from indicated repetitive elements in vehicle- and uridine (100 μM)-treated HGPS hMSCs (P10) at P2 post treatment. Right, RT-qPCR analysis of transcripts from indicated repetitive elements in vehicle- and uridine (100 μM)-treated hPMSCs (P13) at P2 post treatment. Data presented as the mean ± SEM (Two-tailed unpaired Student's *t* test). *n* = 4. **f** Left, immunostaining of γH2A.X and 53BP1 in vehicle- and uridine (200 μM)-treated WS hMSCs (P5) at P2 post treatment. Middle, immunostaining of γH2A.X and 53BP1 in vehicle- and uridine (100 μM)-treated HGPS hMSCs (P10) at P2 post treatment. Right, immunostaining of γH2A.X and 53BP1 in vehicle- and uridine (100 μM)-treated hPMSCs (P13) at P2 post treatment. Dashed lines indicate the nuclear boundaries of the γH2A.X and 53BP1 double positive cells. Data are presented as the mean ± SEM. (Two-tailed unpaired Student's *t* test). *n* = 3 biological replicates. Scale bars, 25 μm.

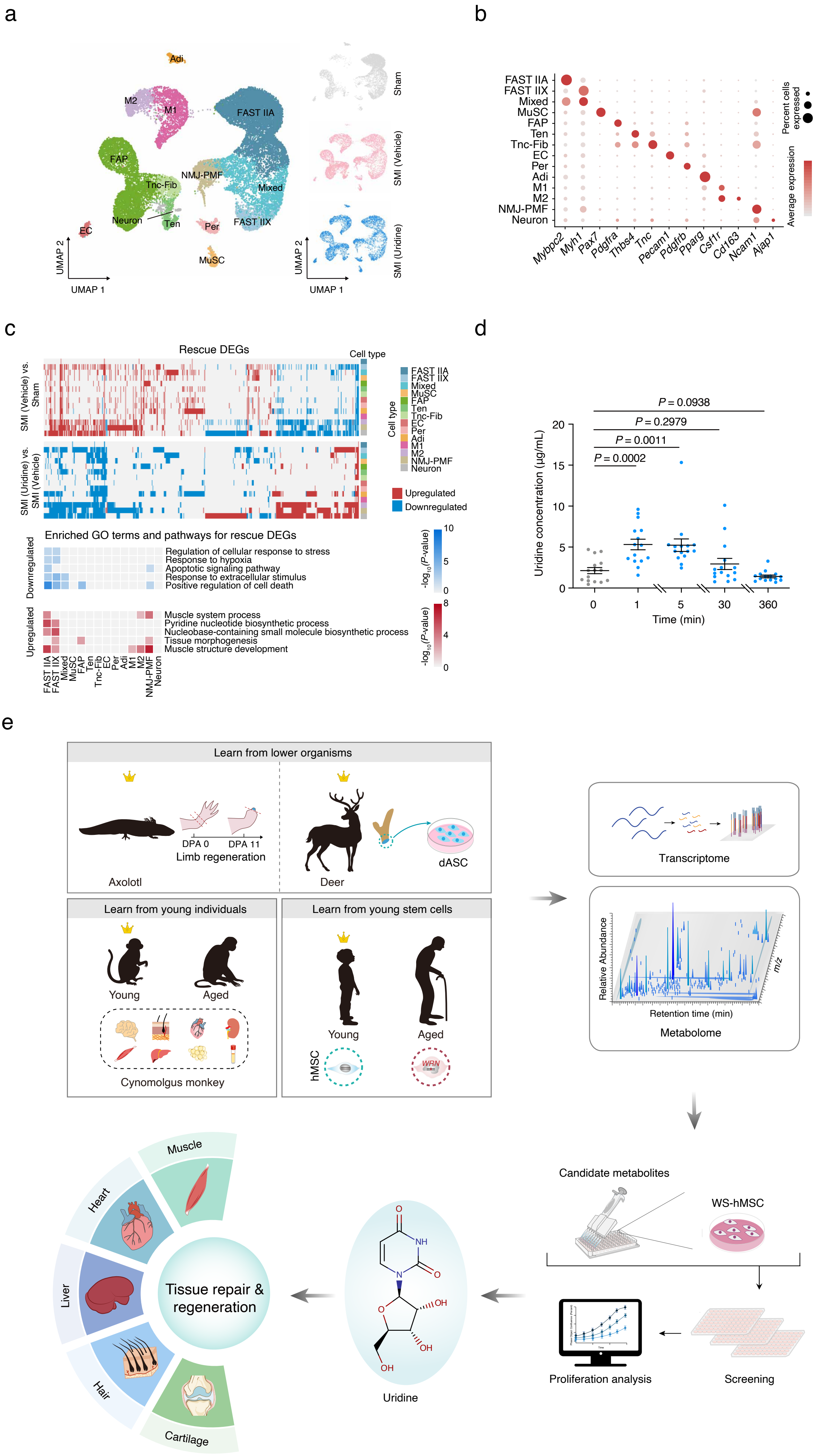

a
Adi
M2
M1
FAST IIA
FAP
NMJ-PMF
Tnc-Fib
Mixed
Neuron
Ten
EC
Per
FAST IIX
MuSC
UMAP 2
UMAP 1
Sham
SMI (Vehicle)
SMI (Uridine)
b
FAST IIA
FAST IIX
Mixed
MuSC
FAP
Ten
Tnc-Fib
EC
Per
Adi
M1
M2
NMJ-PMF
Neuron
Mybpc2
Myh1
Pax7
Pdgfra
Thbs4
Tnc
Pecam1
Pdgfrb
Pparg
Csf1r
Cd163
Ncam1
Ajap1
Percent cells expressed
25
50
75
Average expression
2
1
0
c
Rescue DEGs
Cell type
SMI (Vehicle) vs. Sham
SMI (Uridine) vs. SMI (Vehicle)
FAST IIA
FAST IIX
Mixed
MuSC
FAP
Ten
Tnc-Fib
EC
Per
Adi
M1
M2
NMJ-PMF
Neuron
Upregulated
Downregulated
Enriched GO terms and pathways for rescue DEGs
Downregulated
Regulation of cellular response to stress
Response to hypoxia
Apoptotic signaling pathway
Response to extracellular stimulus
Positive regulation of cell death
-log10(P-value)
10
5
0
Upregulated
Muscle system process
Pyridine nucleotide biosynthetic process
Nucleobase-containing small molecule biosynthetic process
Tissue morphogenesis
Muscle structure development
8
4
0
d
P = 0.0938
P = 0.2979
P = 0.0011
P = 0.0002
Uridine concentration (μg/mL)
20
15
10
5
0
0
1
5
30
360
Time (min)
e
Learn from lower organisms
DPA 0
DPA 11
Limb regeneration
Axolotl
Deer
dASC
Learn from young individuals
Young
Aged
Cynomolgus monkey
Learn from young stem cells
Young
Aged
hMSC
WRN
Transcriptome
Relative Abundance
Retention time (min)
m/z
Metabolome
Candidate metabolites
WS-hMSC
Screening
Proliferation analysis
NH
O
N
OH
Uridine
Tissue repair & regeneration
Muscle
Heart
Liver
Hair
Cartilage

Supplementary Fig. S5. Single-nucleus transcriptomic profile of muscle regeneration model, Related to Fig.4.

a UMAP plots showing the different cell types (left) and cells of muscle tissues from sham mice and mice treated with vehicle or uridine (right). Adi, adipocyte; FAST IIA, type IIA fast-twitch muscle fiber; FAST IIX, type IIX fast-twitch muscle fiber; Mixed, mixed type IIA/ IIX fast-twitch muscle fiber; MuSC, Muscle satellite cell; FAP, fibro-adipogenic progenitor; Ten, Tendon fibroblast; Tnc-Fib, *Tnc*-positive fibroblast; EC, endothelial cell; Per, pericyte; M1, pro-inflammatory macrophage; M2, anti-inflammatory macrophage; NMJ-PMF, neuromuscular junction-postsynaptic muscle fiber. **b** Dot plot showing the representative marker gene in each cell type. **c** Top, heatmaps showing the distribution of DEGs of cell types comparing between SMI (Vehicle) and Sham groups, and SMI (Uridine) and SMI (Vehicle), respectively. Each row represents one cell type, and each column represents one gene. Rescue DEGs are the genes that exhibited the opposite changes upon injury and uridine treatment. Bottom, GO term and pathway enrichment analysis for rescue DEGs. The color keys from white to red or blue indicate $-\log_{10}(P\text{-value})$ from low to high. **d** Pharmacokinetic characteristics of plasma uridine concentrations in mice (n = 15 mice) after intraperitoneal administration (200 μL of 4 mg/mL uridine in 0.9% NaCl). Blood samples were serially taken, and the uridine concentrations were determined by liquid chromatography-tandem mass spectrometry. Each sample was technically repeated for 6 times, and the mean value was taken for statistical analysis. Data are presented as the mean ± SEM (Two-tailed unpaired Student's t test). **e** Schematic diagram showing the working model for cross-species and cross-ages metabolomic analysis and metabolites screening identified uridine as a key metabolite reinforcing regeneration in multiple mouse tissues.

Description of Supplementary Tables

File Name: Supplementary Table S1

Description: Information for overlapping discriminatory metabolites elevated in samples with higher regenerative potentials.

File Name: Supplementary Table S2

Description: Detailed information of metabolites screening assays.

File Name: Supplementary Table S3

Description: The sequences of primers used in this study.

File Name: Supplementary Table S4

Description: List of antibodies used in this study.
